# A comprehensive assessment of water quality in Fayoum depression, Egypt: identifying contaminants, antibiotic pollution, and adsorption treatability study for remediation

**DOI:** 10.1038/s41598-024-68990-8

**Published:** 2024-08-14

**Authors:** Mai Sayed Fouad, Emad Fawzy Mustafa, Mohamed Saad Hellal, Mai Ali Mwaheb

**Affiliations:** 1 Botany Department, Faculty of Science, Fayoum University, Fayoum, 63514 Egypt; 2 Water Management Research Institute, National Water Research Center NWRC, Shubra El Kheima, Egypt; 3 Water Pollution Research Department, National Research Centre, Cairo, 12622 Egypt

**Keywords:** Water quality, Microbial parameters, Antibiotics, Adsorption, *Phragmites australis*, Environmental sciences, Freshwater ecology

## Abstract

This study aimed to assess the current water quality status across various regions within the Fayoum depression by examining water canals, drains, and potential contaminants impacting public health and the local ecosystem. Additionally, an adsorption treatability investigation was conducted on various antibiotics identified during the assessment. Fifteen sampling points were selected across the Fayoum depression, covering surface water bodies and agricultural drainage systems during both winter and summer seasons. Physico-chemical, microbiological, and antibiotic analyses were performed on collected water samples. The water quality parameters investigated included pH, electrical conductivity, total dissolved solids (TDS), total coliforms, fecal coliforms, and concentrations of antibiotics such as ciprofloxacin and tetracycline. The findings revealed significant variations in water quality parameters among different water sources, categorizing them into three types: irrigation canals, polluted canals, and drains. High contamination levels were observed in certain water canals and drains due to untreated sewage and agricultural drainage discharge. Notably, elevated TDS levels (exceeding 1200 mg/L), microbial indicators count (with total coliforms reaching up to 2.3 × 10^6^ CFU/100 mL), and antibiotics (with concentrations of ciprofloxacin and tetracycline exceeding 4.6 µg/L) were detected. To mitigate antibiotic contamination, a Phyto-adsorption treatability study using magnetite nanoparticles prepared with Phragmites australis plant extract demonstrated promising results, achieving complete removal of high antibiotic concentrations with an adsorption capacity of up to 67 mg/g. This study provides updated insights into water quality in the Fayoum depression and proposes a novel approach for addressing antibiotic contamination, potentially safeguarding human and environmental health.

## Introduction

In many regions, water shortages are common problems, especially in the MENA region, where most countries are dry or semi-dry. With an increase in water demand, drought leads to a lack of water over time. These countries must address the problem of water shortages by addressing the challenges they face^1^. The use of non-conventional water is one of the strategies to deal with water shortages^2^. Today, when deciding future directions, the first concern is paying close attention to the challenges of water resources. There are significant differences between various drain water characteristics depending upon the region in the world. The release of drain water is associated with wastewater that, in turn, is a blend of the used water from the domestic sector and the industrial drains^3^. There are significant differences between various drain water characteristics depending upon the region in the world^4^. In the past and in the present, the development of specific sewer systems targeting wastewater from major urban settlements has contributed to improving the quality of surrounding water bodies^5^. Contrary to the pact between technologies and society, during the most recent decades, human activity has implemented anthropogenic changes by releasing an increasing number of pollutants, compromising the balance of environmental reservoirs^6^. The focus is on source control for water quality of centralized storm and wastewater systems because this addresses both climate change and pollution challenges concurrently and is not commonly practiced, particularly in a temperate maritime climatic region^3^. Among these countries, Egypt faces a lack of water resources and the gap between Egypt's freshwater supply and water demand is increasing every year^7^. The main challenges in Egypt's freshwater supply include increased urban demand, changes in land use, and environmental requirements^8^, but climate change has a major impact and Ethiopia's Renaissance Dam is the most important challenge^9^. Recently, Egypt has adopted the same strategy to fill the gap between water supply and demand through non-conventional water resources and good management of current resources. Consequently, regular monitoring of the quality of surface water from the Nile River is now considered to be Egypt's main objective in national policy^10^. Polluted rivers are dangerous not only for human health, who directly consume the water, but also because crops are irrigated by these rivers^11^.

Fayoum, as one of the Egyptian governorates, is a mini model of Egypt's water status, as it is an inland closed drainage basin located in the arid region characterized by dry and hot conditions. Fayoum depression is irrigated by the Nile River from Bahr Yousef canal through Lahon regulator. However, it suffered an imbalance in its water supply and demand due to the progressive expansion of the redevelopment of new desert lands. Water shortages have resulted in inconsistency in the distribution of water in irrigation channels. In addition, there are other challenges related to water, including the ability to compensate for the available freshwater shortage, controlling canal contamination and pollution loads in the drains, and controlling waste volumes of drainage water effluents in Qarun Lake and Wadi El-Raiyan^12^. All excess water from agriculture, including treated and untreated domestic wastewater, is usually collected in drainage channels.

However, in some areas, wastewater from sewage treatment plants is released directly into irrigation channels^13^. Subsequently, most of the water flows into two major channels. El-Wadi and El-Bats, which together with smaller channels, eventually flow to Qarun Lake. Several studies were carried out on regular water quality monitoring activities to investigate seasonal variations in various quality parameters, including agricultural, domestic, and industrial pollutants in Fayoum depression^14–17^. These studies focused on the quality of the water in the main drains (El Bats and El Wadi) or the Qarun lake. However, the data available to cover both drainage and fresh water at different sites in the depression are little.

In addition, another challenge facing water quality is the growth of unwanted plants such as *Phragmites australis* in canals and drains, which requires regular clearance, resulting in contamination with residual agricultural waste. However, these unwanted plants could be useful if used properly in the phytoremediation of polluted drains. The roots and rhizomes of plants could be a good source of bionanomaterial phytosynthesis for the removal of hazardous micropollutants such as antibiotics from water^18^. Yan et al.^19^ investigated the uptake of antibiotics from *Eichhornia crassipes* in the seedling and mature stages under hydroponic conditions. Furthermore, Hosny et al.^20^ used aqueous extracts of *Phragmites australis* as alternative reducing agents to conventional chemicals for the synthesis of gold nanoparticles. *Phragmites australis* can also be used for the synthesis of magnetite nanoparticles (NPs). Among magnetite NP synthesis methods, the reduction of ferric or ferrous iron with plant-derived bioactive compounds has gained notable interest attributed to its cost-effectiveness and eco-friendliness^21^. The process involves using metabolites such as carbohydrates, glycosides, alkaloids, flavonoids, saponins, phenols, proteins, quinine, steroids, and tannins as reducing and stabilizing agents in nanomaterial fabrication^22^. Consequently, *Phragmites australis,* commonly found in tropical and warm regions, such as Fayoum depression on the banks of drains and canals, served as a reducing and stabilizing agent for magnetite NP synthesis.

The presence of antibiotics in surface water and drainage water is a result of several contributing factors. These include direct discharge of pharmaceutical manufacturing effluents, agricultural runoff containing antibiotic-laden animal manure, and incomplete removal of antibiotics during wastewater treatment processes^23^. Furthermore, antibiotics can enter the aquatic environment through excretion by humans and animals, as well as improper disposal of unused medications. Studies conducted around the world have detected a wide range of antibiotics in surface water and drainage water samples^24^. For example, commonly used antibiotics such as sulfonamides, fluoroquinolones, tetracyclines, and ciprofloxacin have been found in surface waters in diverse geographical regions, including urban, rural, and remote areas^24–27^. The concentrations of these antibiotics vary, but even trace amounts can exert selective pressure on bacterial communities, potentially leading to the development of antibiotic resistant strains. This raises concerns about the potential human exposure to antibiotic residues through the consumption of contaminated aquatic organisms. To mitigate these concerns, it is imperative to develop and implement effective methods for the removal of antibiotics from surface and drainage water. Fewer studies have discussed nanoparticle biosynthesis using plant extracts for the removal of or antibiotics from water bodies.

This study aims to comprehensively monitor and evaluate the water quality of various canals and drains within the Fayoum depression. It seeks to identify and determine the potential sources of pollution impacting these water bodies and to monitor the fate and impact of these pollutants on the quality of surface water. The research will provide an updated assessment of water quality in the Fayoum depression across different seasons, specifically during winter and summer, over the period from 2020 to 2022 to overcome lack of recent and comprehensive data on the water quality of the Fayoum depression. Additionally, the study includes an investigation into the treatability of antibiotic pollutants in water using phyto-synthesized magnetite nanoparticles. These nanoparticles are derived from the aqueous extract of Phragmites australis, a plant commonly found growing along the banks of drains and canals in the region.

## Materials and methods

### Sampling sites

**Figure 1:**
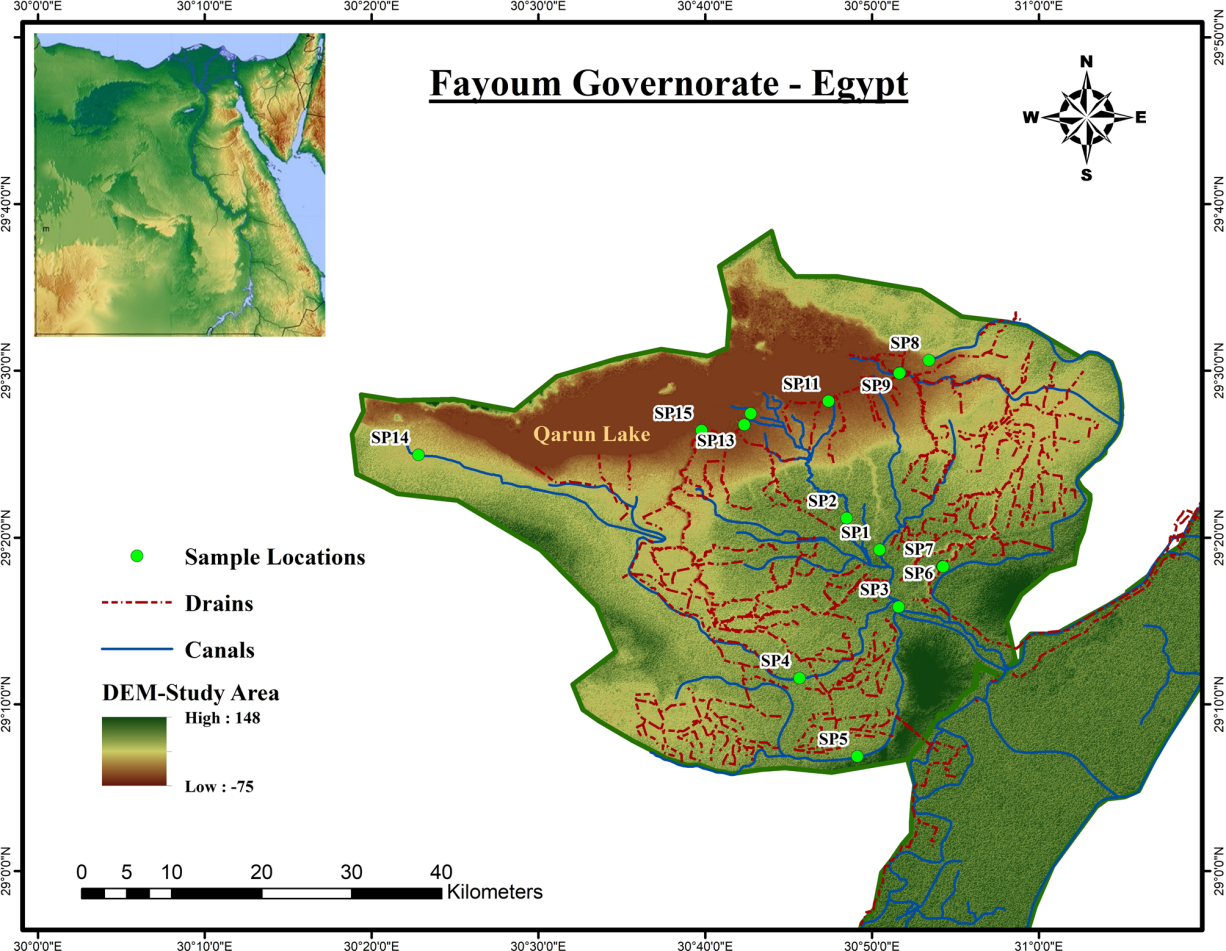
Fayoum Governorate location map including sampling points sites.

Fayoum depression is positioned between latitudes 28° 55′ N–29° 40′ N and longitudes 29° 55′ E–31° 50′ E, covering an area of 6068 km^2^ and lying approximately 95 km southwest of Cairo. It is bordered by desert on all sides except for its south-eastern corner, which connects it to the Beni-Sueif governorate (Fig. 1). Fayoum agriculture is dependent on Nile water, with its share amounting to approximately 2.56 billion cubic meters annually along with minor contributions from groundwater and negligible amounts of rainwater. Historically, the water quality in this area has been under threat due to various sources of pollution, including agricultural runoff, industrial discharges, and urban wastewater. Monitoring and improving the water quality in Fayoum is crucial for ensuring the sustainability of its agricultural productivity, safeguarding public health, and protecting local biodiversity. Recent studies have highlighted the presence of various contaminants, including heavy metals, organic pollutants, and antibiotics, which necessitates ongoing research to develop effective mitigation strategies.

Water samples were collected from 15 different sites to cover main irrigation canals and the drainage network throughout the governorate. The descriptions of the sample’s sites are indicated in Table 1. While the sample locations are illustrated in the map created using the software Arc GIS V. 10.8 (https://www.esri.com/en-us/arcgis/products/arcgis-desktop/resources) as indicated in Fig. 1 . These points include locations upstream and downstream of major pollution sources, such as industrial effluents and agricultural runoff, as well as sites near residential areas and within protected natural habitats. This selection ensures that the data collected reflects the diverse conditions and pollution levels throughout the region, enabling a thorough analysis of water quality dynamics and pollution sources. The samples were collected periodically during the summer and winter of the years 2020, 2021 and 2022. Sampling was carried out according to standard methods for the examination of water and wastes^28^**.** All samples collected for chemical, bacteriological, or microbial analysis were stored in an ice-collection box at 4 ± 2 °C and immediately delivered to the laboratory for analysis.

**Table 1:** Monitoring of different sites of Fayoum Canals and Drains.

Samples Group	Sample code	Sample Name	Location coordinates		Surface water direction	Description
			Latitudes N	Longitudes E		
A	SP1	Bahr Sonores canal	29.32122	30.84103	North	Main canal in Fayoum city
	SP2	Bahr Sanhor canal	29.35284	30.808	West	Branch from Sonores canal
	SP3	Hassan Wasef Canal	29.26411	30.85996	West	Starting point of SP4 and SP5
	SP4	El Nazla canal	29.19304	30.76095	West	End of El Nazala canal
	SP5	Bahr El Gharaq canal	29.11469	30.81871	South	End of El Gharaq canal
	SP6	Bahr Whabi canal	29.30469	30.904	North	Starting point of Bahr Wahbi canal
C	SP7	El Bats drain	29.30462	30.90435	North	Starting point of El Bats drain
B	SP8	Bahr Whabi canal	29.51045	30.89033	Northeast	Connection of canal with El Bats drain
C	SP9	El Bats drain	29.49823	30.86151	Northeast	End of El-Bats drain
B	SP10	Bahr EL Wati canal	29.49751	30.86086	West	Connection of the canal with Tanhala drain
C	SP11	El Qatea drain	29.46963	30.78994	North	End of the drain before lake Qarun
B	SP12	El Zaideya canal	29.45731	30.71228	North	End of the canal before lake Qarun
C	SP13	El Zaideya drain	29.4461	30.70578	North	End of the drain before lake Qarun
B	SP14	Bahr Qarun canal	29.41589	30.38034	West	End of the canal before lake Qarun
C	SP15	El Wadi drain	29.44028	30.66324	North	End of the drain before lake Qarun

Also, the choice of collection sites based on the assessment of water bodies at the starting points and at the combination with other water bodies that could affect the water quality of these water. From Table 1, samples from SP1 to SP6 are irrigation water samples of starting and ending points of main canals in Fayoum (6 irrigation canals). These canals didn’t contain any combining or mixing point with any source of wastewater or drainage water. Accordingly, these sampling points (SP1–SP6) were evaluated as non-polluted water and could be identified as Group A. The other sampling points from irrigation sub-canals were SP8, SP10, SP12 and SP14 and these points were selected as they represent mixing with other drainage water and sewage water from treatment plants. For example, SP8 was selected after combination of SP7 (El Bats drain) and SP6 (Bahr Whabi canal) while SP10 is receiving polluted water from Tanhala drain and effluent of anaerobic wastewater treatment plant. These points evaluated as group of polluted irrigation canals and identified as Group B.

The sampling points SP7, SP9, SP11, SP13 and SP15 are selected as points representing different drains in the governorate. These points are also polluted as some sewage treatment plants discharge the wastewater into this drainage. SP7, SP9 represents the beginning and the end of EL Bats Drain which is the longest and main drain in the governorate (51 km long) and it discharged in Qaroun Lake. This drain is receiving effluents from small agricultural drains and effluents from 28 sewage primary treatment plants. SP11, SP13 and SP15 represent the end of El Qatea, El Zaideya and El Wadi drains. These points were identified as Group C. Accordingly, the evaluation of each group was done separately in terms of physicochemical and microbiological characterization.

### Analytical methods procedures

Field parameters (pH, electric conductivity, and dissolved oxygen) were measured in situ using the multiprobe system, model Hydra Lab-Surveyor, and rechecked in laboratory using the following bench-top equipment to ensure data accuracy. Laboratory analyses were carried out according to standard methods for examination of water and wastes^28^. The physico-chemical analysis included pH, electric conductivity (EC), total dissolved solids (TDS), chemical oxygen demand (COD), biological oxygen demand (BOD), ammonia, alkalinity, anions and cations (sulfate, nitrite, nitrate, chloride, phosphate, sodium, calcium, magnesium, potassium,) and heavy metals.

The pH values of the collected field samples were determined using bench-top pH/ISE meters, ORION model 7l0A. EC was measured using ATC bench electric conductivity meters, HANNA, model HI 8820. TDS were analyzed via gravimetric method. Major anions and cations were determined using ion chromatography (IC) (model DX-600, USA). Colorimetric determination of ammonia was achieved using Nessler solution major heavy metals (Cu, Fe, Mn, Pb, Zn, Ni, and Cd) were quantified using Inductively Coupled Plasma-Emission Spectrometry (ICP-ES) with an Ultra Sonic Nebulizer (USN), model Perkin Elmer Optima 3000. Bacteriological analyses included total coliforms (TC), fecal coliforms (FC), and fecal streptococci (FS) using the membrane filtration technique in accordance with standard methods Nos. 9222B, 9222D, and 9230C on M-Endo agar LES, M-Fc agar, and M-Enterococcus agar media respectively. All media used were acquired in a dehydrated form, Difco-USA. Results were recorded as colony forming unit (CFU/100 ml). Results were recorded as colony forming unit (CFU/100 ml). Algae was determined according to phytoplankton counting technique method (00200G) (APHA,2023). The total algae count was recorded using microscope method using Olympus X3 microscope, (Olympus Corporation, Tokyo, Japan). Pour plate method was also used for isolation of fungi using Potato dextrose agar (PDA) (Difco, USA) as selective media for molds^29,30^.

The determination of antibiotics concentration in terms of ciprofloxacin (CIP) and tetracycline (TETC) was carried out using High Performance Liquid Chromatography (HPLC) and UV/visible spectroscopy Shimadzu UV-1800. HPLC method Dionex Ultimate 3000 UHPLC equipped with a pump, on-line vacuum degasser, auto-sampler, Peltier column oven, and UV–Vis detector (Perkin Elmer Series 200 HPLC system, Norwalk, USA) was used for low concentrations detection. The stationary phases used were reversed phase columns like XBridgeR HPLC RP- C18 (4.6 × 250 mm, 5 μm). Mobile phase, consisting of Acetonitrile ACN:H_2_O (75:25, v/v) as the organic components and pH adjusted water with either 85% Formic acid FA, 85% Ortho-phthalaldehyde OPA, or Trifluoroacetic acid TFA or 50 mM KH_2_PO_4_ buffer, pH adjusted with 85% OPA (pH 2.0–3.0) as the buffered aqueous components, pumped at various flow rates in the range of 1.0–2.5 mL /min were evaluated.

### Adsorption treatability study

Batch adsorption experiments were conducted to study the kinetic parameters including reaction time, antibiotic concentrations, and sorbent dose for the removal of CIP and TETC from aqueous solution. The sorption study for each parameter was conducted in 250-mL conical flask containing 100-mL of solution. The sorption time was carried out for a time from 0 to 300 min and the antibiotic concentration tested were 2.5, 10, 25, and 30 mg/L for CIP and 25, 50, 75, 100, 150 and 200 mg/L for TC. The dose of nanocomposite varied between 0.5, 1, 1.5 and 2 g /L.

For each experiment, a 100 mL of solution containing TC and CIP was brought into contact with prepared nanocomposite in a conical flask, then shaking uniformly by orbital shaker (Stuart S1600C shaker) at 200 rpm. After each experiment the mixture is filtered from the nanocomposite and the residual concentration of CIP or TETC was determined before, after treatment and was analyzed by UV/visible spectrophotometer. Full description of nanocomposite preparation and characterization and the adsorption study is illustrated in supplementary file.

### Statistical analysis

Each measurement was made in triplicate to generate the data, which is displayed as the mean standard deviation. An analysis of variance (ANOVA) was conducted to assess the statistical significance of the data, and *P* < 0.05 was considered statistically significant.

## Results

The different water quality characteristics were evaluated to generate a comprehensive index showing the present state of chemical contamination of various constituents of natural waters in Fayoum Depression. More than seventeen different parameters were measured to obtain a good overview of the present state of water quality in the study area. These parameters covered both the inorganic organic and microbiological contamination loads present in the water samples. From the experimental results, it is clearly concluded that Fayoum water was already represented by a contaminated aquifer system with a different variety and large amounts of contaminants. According to identified parameters, some organic pollutants, which are emerging organic contaminants such as antibiotics and pharmaceuticals, are also present in the water of Fayoum depression. As shown in Table 1, the water samples were collected from drainage and irrigations canals and each type of water has different regulatory guidelines and limits to water quality inside the water body^31^. These National regulatory limits are presented in Supplementary Table 1S.

### Evaluation of physico-chemical analysis

**Figure 2:**
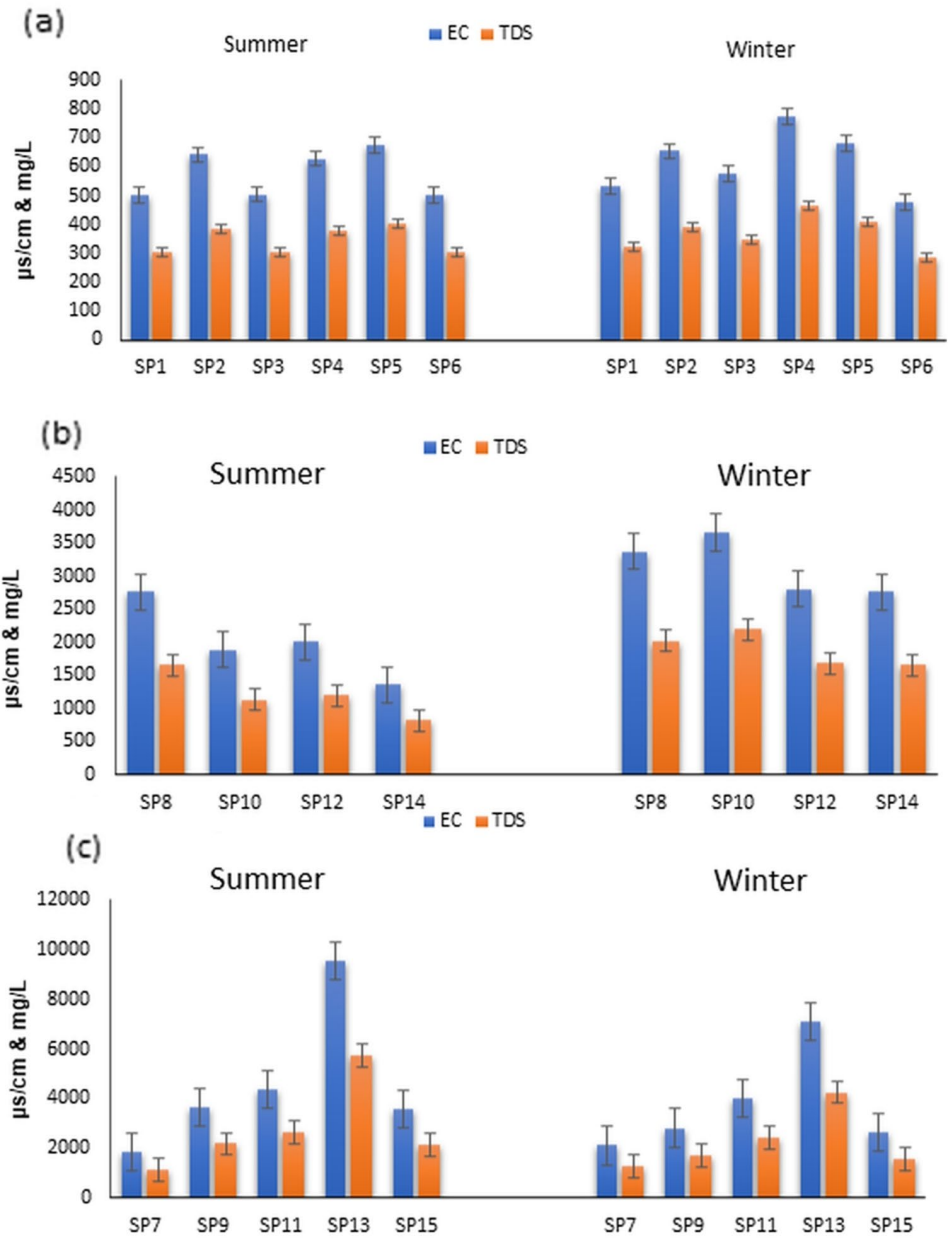
Variation of EC and TDS during summer and winter in samples Groups A, B and C.

**Figure 3:**
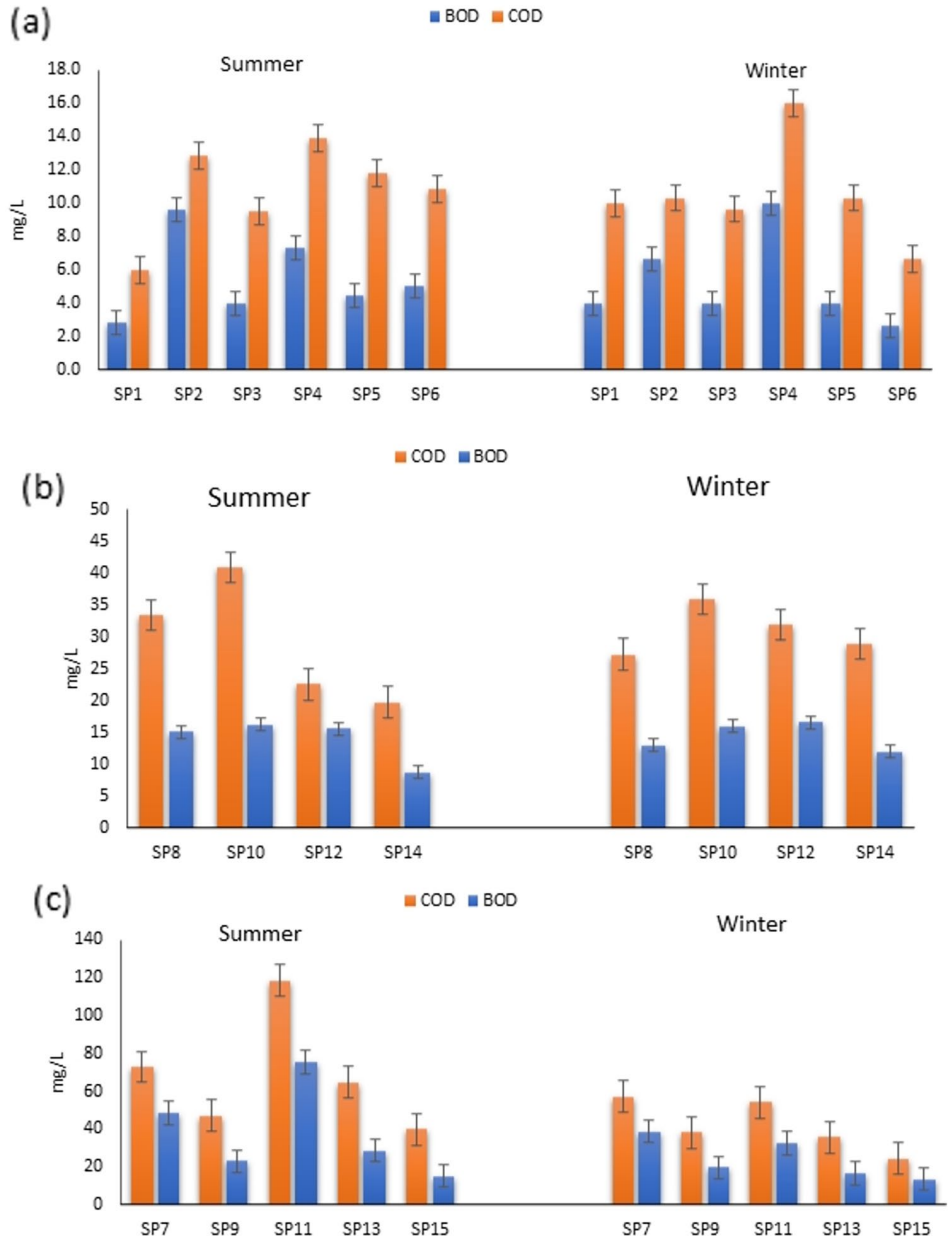
COD and BOD levels in different sampling sites (Group A, B, C) during summer and winter.

**Figure 4:**
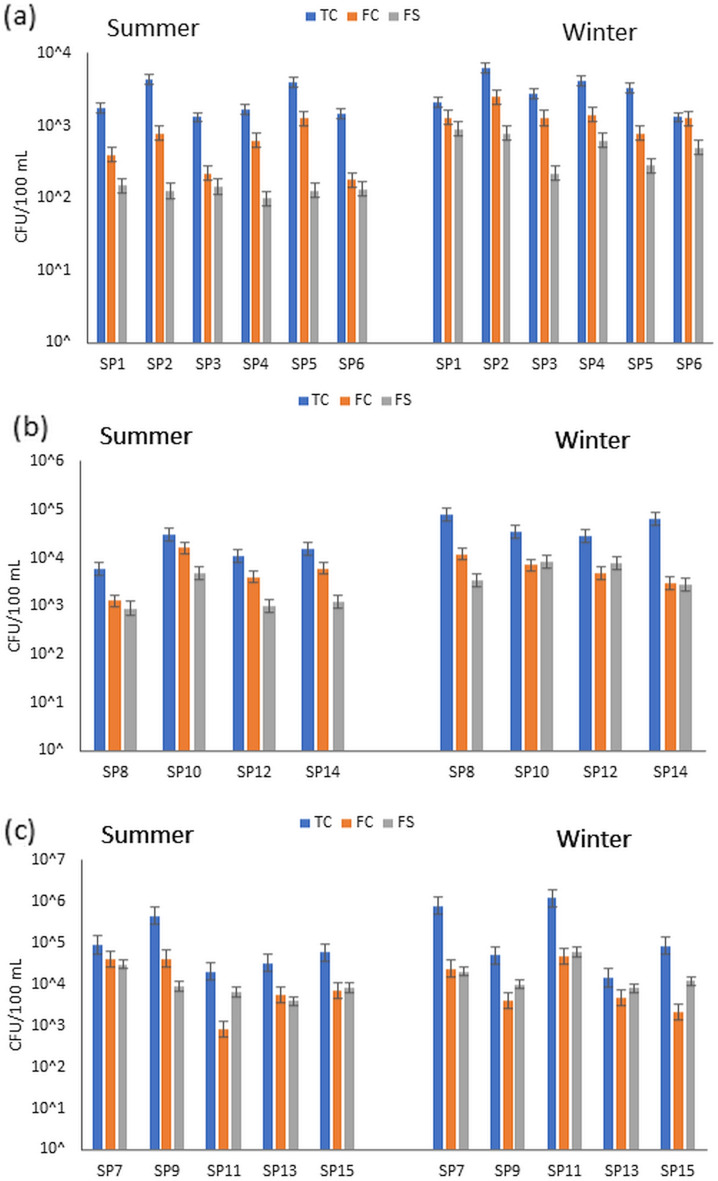
Total coliform (TC), fecal coliforms (FC) and fecal streptococci (FS) for all collected samples during winter and summer.

**Figure 5:**
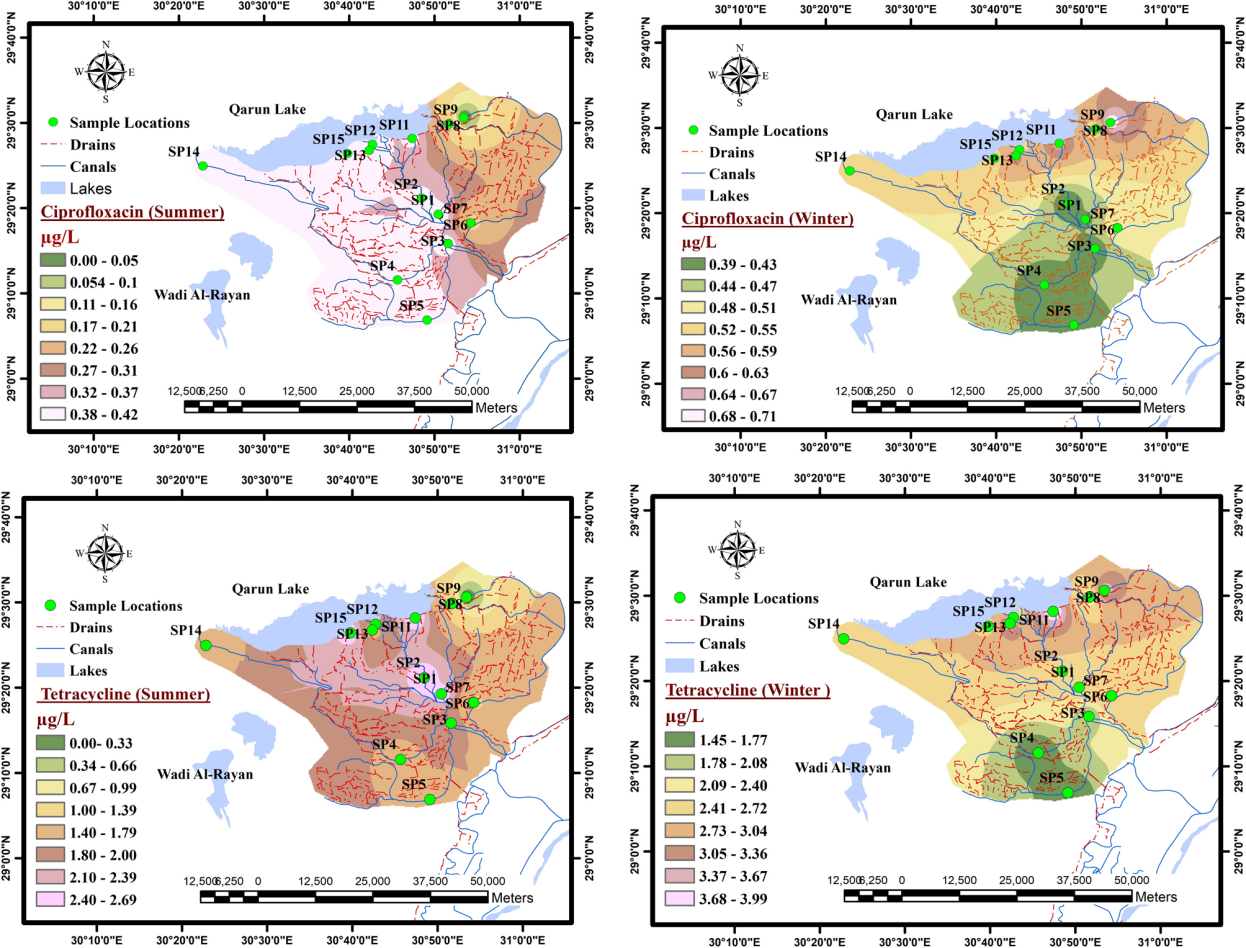
Tetracycline and Ciprofloxacin variation in different water samples collected during winter and summer.

The physical and chemical examination of water samples taken from various locations in Groups A, B, and C in the summers and winters of 2020, 2021, and 2022 was analyzed statistically to determine the level of differences, as detailed in Supplementary Tables 2S–4S. The detailed assessment of variations in different parameters is presented in Figs. 2, 3, 4 and 5 and Supplementary Figs. (4S–6S).

The findings from the testing of water samples from various irrigation canals and drains in summer and winter for Groups A, B, and C are displayed in Fig. 2. The results in Table 2S indicated that the pH levels averaged between 7.7 and 8.1, with no disparities between the two seasons, and the highest pH value was in SP1, indicating a slightly alkaline nature. The analysis of the EC and TDS values for freshwater samples (SP1–SP6) can be seen in Fig. 2a, where the EC values were found to range from 500 ± 12 to 675 ± 15 μs/cm in summer and increase to 774 ± 76 μS/cm in winter. Similarly, TDS ranged from 300 ± 24 to 400 ± 19 mg/L in summer and increased to a range of up to 465 ± 48 mg/L in winter. The mean values of EC and TDS during winter closure were 615 ± 115 μs/cm and 369 ± 68 mg/L, respectively, while in the summer, they averaged 431.9 ± 91 μs/cm and 260 ± 54 mg/L, respectively. The higher values of EC and TDS during the winter closure as compared to summer indicate the significant impact of reduced flow on water salinity. This reduction in water flow in winter results in the concentration of dissolved salts, exceeding that of the summer levels.

**Table 2:** Kinetic and isotherm parameters for adsorption of TETC and CIP antibiotic.

Parameters	TETC	CIP
qe_exp_ (mg/g)	67.7	6.25
Pesou-frist order model		
qe_cal_ (mg/g)	64.1	2.93
k_1_ (1/min)	0.052	0.113
R^2^	0.985	0.78
Pesou-frist order model		
qe_cal_ ((mg/g)	56.4	5.99
K_2_ (1/min)	0.12	0.142
R^2^	0.808	0.989
Langmuir		
qm (mg/g)	63.9	7.13
K_L_ (L/mg)	0.55	0.13
R_L_	0.064	0.038
R^2^	0.83	0.85
Freundlich		
K_F_ (L/mg)	0.78	0.72
n	1.64	1.34
R^2^	0.99	0.95

Upon analyzing water samples from irrigation canals in Group B (SP8, SP10, SP12, and SP14), it was observed that there was a significant increase in TDS and EC levels, with the values being more than double that of Group A. For instance, SP8 showed the highest average values in the summer, recording 915 ± 45 mg/L TDS and 1655 ± 115 μs/cm EC. This location represents the Bahr Wahbi canal after it is mixed with the drain of EL Bats, which contains sewage and agricultural drainage water. Similarly, SP10 displayed higher values in the winter, with 2195 ± 63 mg/L and 3660 ± 142 μs/cm for TDS and EC, respectively, indicating significant pollution. Even though SP12 and SP14 did not mix with any drains, they still showed high TDS concentrations, reaching 1650 ± 41 mg/L in the winter. Furthermore, the TDS concentrations were higher in winter than in summer, with all the samples from the determined sites having an average of 1856 ± 458 mg/L and 3109 ± 763 μs/cm compared to 1204 ± 415 mg/L and 2007 ± 691 μs/cm (Table 3S), which indicates the high contamination of these canals with drainage and sewage water.

Group C of water samples (SP7, SP9, SP11, SP13, and SP15) are different drain sites in Fayoum depression. These drains are EL Bats (SP7, SP9), EL Qatea (SP11), El Zaideya (SP 13) and El Wadi (SP15). These drains receive agricultural drainage and untreated sewage water. For example, there are more than 28 wastewater treatment plants that discharge their effluents, which are treated only primary, into El Bats drain (SP7 and SP9). As shown in Fig. 2c, the TDS and EC concentrations in all the drains are extremely high and ranged between 12,051 ± 58 mg/L and 5900 ± 45 mg/L. In addition to seasonal TDS analysis, the components of these salt content in water were evaluated in terms of elements of Na^+^, Ca^2+^, Mg^2+^, K^+^, Cl^−^ and SO_4_^2−^. The results in supplementary Figs. 3S and 4S showed that Na^+^, Cl^−^, Ca^2+^, and SO_4_^2-^ have similar concentration patterns.

The assessment of organic matter in water was carried out through the evaluation of COD and BOD, which are then presented in detail in Fig. 3a–c. The findings indicate that during the winter season, group A's irrigation water had an average COD concentration of 10.5 ± 3.4 mg/L at all sites, except for SP4 which had a higher average value of 16 ± 2.4 mg/L. Similarly, the average BOD concentration was around 4 ± 1.2 mg/L for most areas, but SP4 had a notably higher concentration of 9.1 ± 2.1 mg/L (Fig. 3a). The study also revealed that the mean COD value was 12.7 ± 2.3 mg/L, with SP4 showing a higher presence of organic compounds in the water and an average BOD concentration of 8.5 ± 1.8 mg/L. The monitoring of other polluted canals (group B) showed clear contamination with organic matter, with COD ranging from 20 to 40 mg/L and BOD concentration ranging from 10 ± 1.5 to 16 ± 3.1 mg/L. Notably, SP10 had the highest concentration in both summer and winter. Group C also demonstrated high concentrations of COD and BOD in most sampling points during the summer compared to the winter season. SP11 had the highest average values of 119.6 ± 21 mg/L for COD and 75.2 ± 10.5 mg/L for BOD during the summer. This data is crucial in understanding the presence and distribution of organic matter in the water, with implications for water quality and environmental health.

### Microbiological characterization

The results of bacteriological analyses are of utmost importance when it comes to evaluating and determining the quality of water in various aspects and applications. It is through these analyses and assessments that we can comprehend the condition and characteristics of the water in question. In reference to the data presented in Fig. 4a, it becomes apparent that the total coliform (TC) values tend to be higher during the summer season as compared to the winter season for Group A. Specifically, during the summer months, the TC concentrations range from 3.8 × 10^2^ to 4.6 × 10^3^ colony forming units per 100 ml (CFU/100 mL), whereas during the winter, the TC concentrations lie between 3.9 × 10^3^ and 4.9 × 10^3^ CFU/100 mL. Similarly, the fecal coliform (FC) and fecal streptococcus (FS) levels also exhibit a similar trend of being higher during the summer season compared to the winter season. This pattern of elevated microbial indicators is observed not only in Group A but also in Group B, with higher bacterial counts during the summer as opposed to the winter. It is crucial to note that all sources of irrigation water, particularly those utilized as drinking water in rural areas without proper treatment, invariably contain noticeable levels of TC, FC, and FS. Moreover, when it comes to the drainage waters in Group C, the escalated levels of bacterial contamination are primarily associated with effluents originating from sewage and wastewater treatment plants, as well as agricultural runoff. It is evident that the sampling site of SP9, which focuses on drain water, showcases the highest concentrations of TC and FC during the summer season, with measured values of 2.3 × 10^6^ and 4.9 × 10^5^ CFU/100 mL, respectively. Conversely, during the winter season, the sites of SP7 and SP11 demonstrate substantial levels of microbial pollution, reaching up to 2.1 × 10^6^ CFU/100 mL for TC, thus signifying the presence of sewage within the water samples. These significant findings and crucial insights can be observed in Fig. 4b, further reinforcing the importance of proper water treatment and management practices to ensure the provision of clean and safe water for various applications and purposes.

In addition to bacterial populations, the study also examined the presence of algae and fungi in irrigation and drainage water. Data collected over a three-year period (Table 5S) revealed substantial variations in fungal abundance between the two seasons, shedding light on the complex interplay of environmental factors that affect fungal communities. During the summer season, the fungal counts in non-polluted canal water (SP1-SP6) were significantly higher (average from 12 to 34 CFU/mL) compared to the winter season (from 6 to 16 CFU/mL). Same behavior was found in samples from polluted canals and drains (SP7-SP15) with higher fungal counts values. Also, the results showed that the group A samples contain the highest algal counts than polluted water in groups B and C as indicated in supplementary Fig. 6S. During the winter and summer months, the total algal cell counts experienced a substantial surge, reached range between 8.5 × 10^5^ cells/L and 1.35 × 10^6^ cells/L. Polluted canals (group B) showed similar results with average cell count of 7.6 × 10^5^ cells/L while the drainage water (group C) exhibited the lowest algal count in summer (5.45 × 10^5^ cells/L) and winter (5.2 × 10^5^ cells/L). Furthermore, the composition of total algae exhibited variation in response to distinct water quality parameters in the different canals and drains. In areas with higher inputs of organic matter, such as those receiving sewage discharges and agricultural runoff, diatoms and green algae predominated, constituting up to 60% of the total algal community.

### Antibiotics analysis

In this comprehensive physicochemical characterization of irrigation water, an additional aspect of significant concern was the presence of antibiotics, specifically ciprofloxacin and tetracycline. Antibiotics are known to be important pharmaceutical compounds used in human and veterinary medicine to combat bacterial infections. However, their widespread use has raised concerns about their occurrence in the environment, including water sources used for irrigation. Data regarding the assessment of residual antibiotic concentrations in various Egyptian water sources is limited and does not align with the growing global interest in this research area.

Figure 5 depicts a distribution map was created using the software Arc GIS V. 10.8 (https://www.esri.com/en-us/arcgis/products/arcgis-desktop/resources) with inverse distance weighted interpolation for the concentrations of two prevalent antibiotics, ciprofloxacin (CIP) and tetracycline (TETC), as detected in water samples gathered from different sampling locations within groups A, B, and C during both winter and summer seasons. The chosen antibiotics were found in all samples, and it was observed that elevated concentrations of these antibiotics were primarily present in samples obtained from polluted canals (Group B) and drainage water (Group C), where sewage and household wastewater are discharged into these water bodies. The concentrations of the detected antibiotics show temporal variations with higher levels in winter season as relevant to those recorded in summer season as CIP and TETC concentrations were ranged from 0.3 to 0.7 μg/L and from 1.4 to 3.99 μg/L in winter while in summer were from 0.04 to 0.42 μg/L and from 0.28 to 2.6 μg/L, respectively.

### Adsorption of antibiotics on Phyto-magnetite

As illustrated in characterization tests, the synthesized Phyto-magnetite nanocomposite has been successfully prepared according to TEM, FTIR, and XRD analyses. The adsorption capabilities of TETC and CIP were estimated as model antibiotic compounds found in wastewater.

#### Effect of contact time

**Figure 6:**
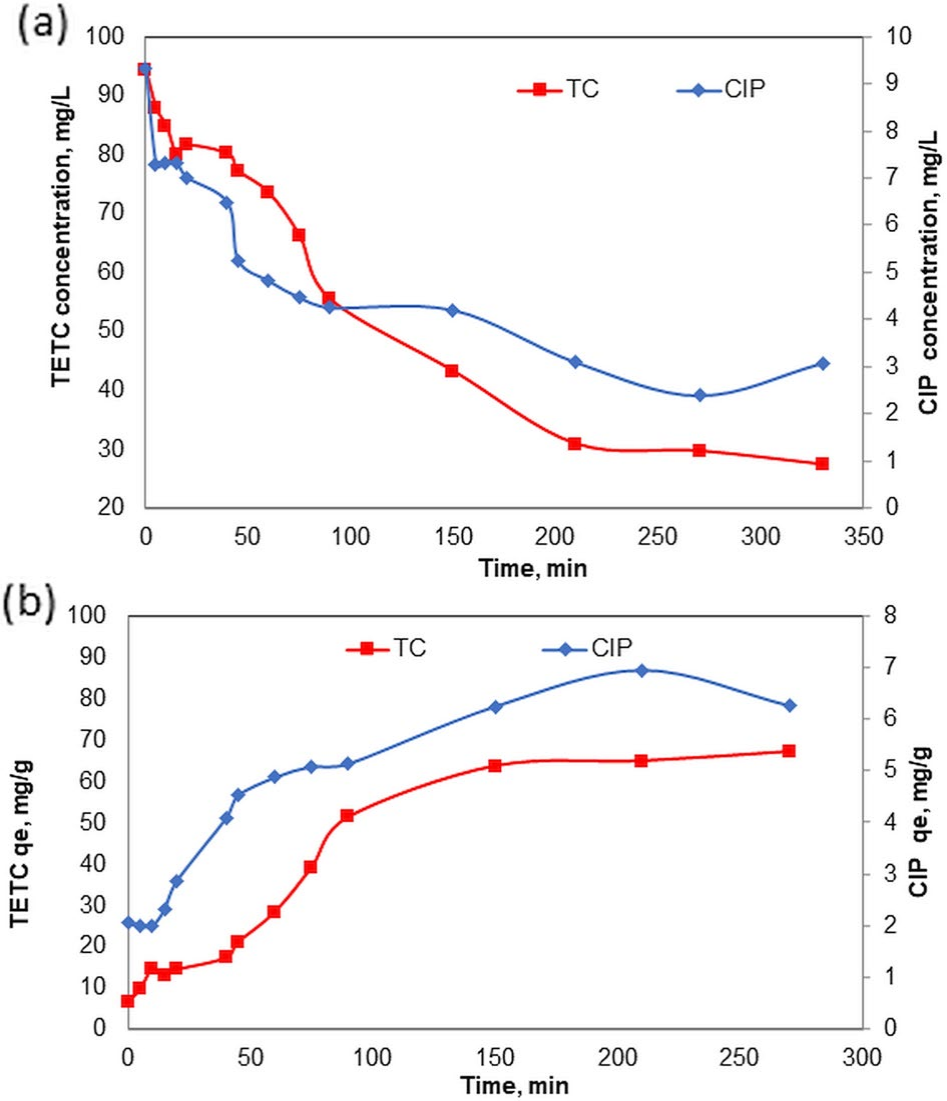
Effect of contact time on (**a**) reduction of TETC and CIP (**b**) adsorption capacity of Phyto-magnetite for TETC and CIP.

The influence of the duration of contact on the adsorption of 100 mg/L Tetracycline (TETC) and 10 mg/L Ciprofloxacin (CIP) by nanocomposite materials at pH 7 and the adsorbent dosage of 1g/L was investigated. The results of this study are illustrated in Fig. 6a and b. The data suggest that both the adsorption capacity and the percentage of TETC removal exhibit significant increases during initial contact periods, decelerating after approximately 200 min and nearing equilibrium around 240 min. The removal of TETC removal was detected to be greater than 70% in 200 min; The removal rate of TETC steadily decreased until the contact period of 330 min (the optimum contact period), after which it remained constant as shown in Fig. 6a. Therefore, the optimal contact time of 300 min was chosen for other sorption studies. The adsorption behavior with time in the case of CIP was quite different. The removal of CIP increased significantly in a regular pattern up to 90 min with a removal rate of 55%. The adsorption rate starts to be slower during 60 min, and only 3% increase in removal was achieved, as the removal rate was 57% at 150 min. After another 120 min, the removal rate is reduced by 20%. In Fig. 6b, the data on the uptake capacity of TETC and CIP from the solution with time are shown. The maximum adsorption capacity was achieved from 150 min as the qe value reached 64.3 mg/g reaching 67 mg/g at 330 min. The adsorption capacity obtained for TETC at 330 min is in agreement with the study by^32^ who obtained the qe value 72 mg/g for the removal of TETC using Phyto -zerovalent iron nano-particles.

#### Effect of sorbent dosage and antibiotic concentration

**Figure 7:**
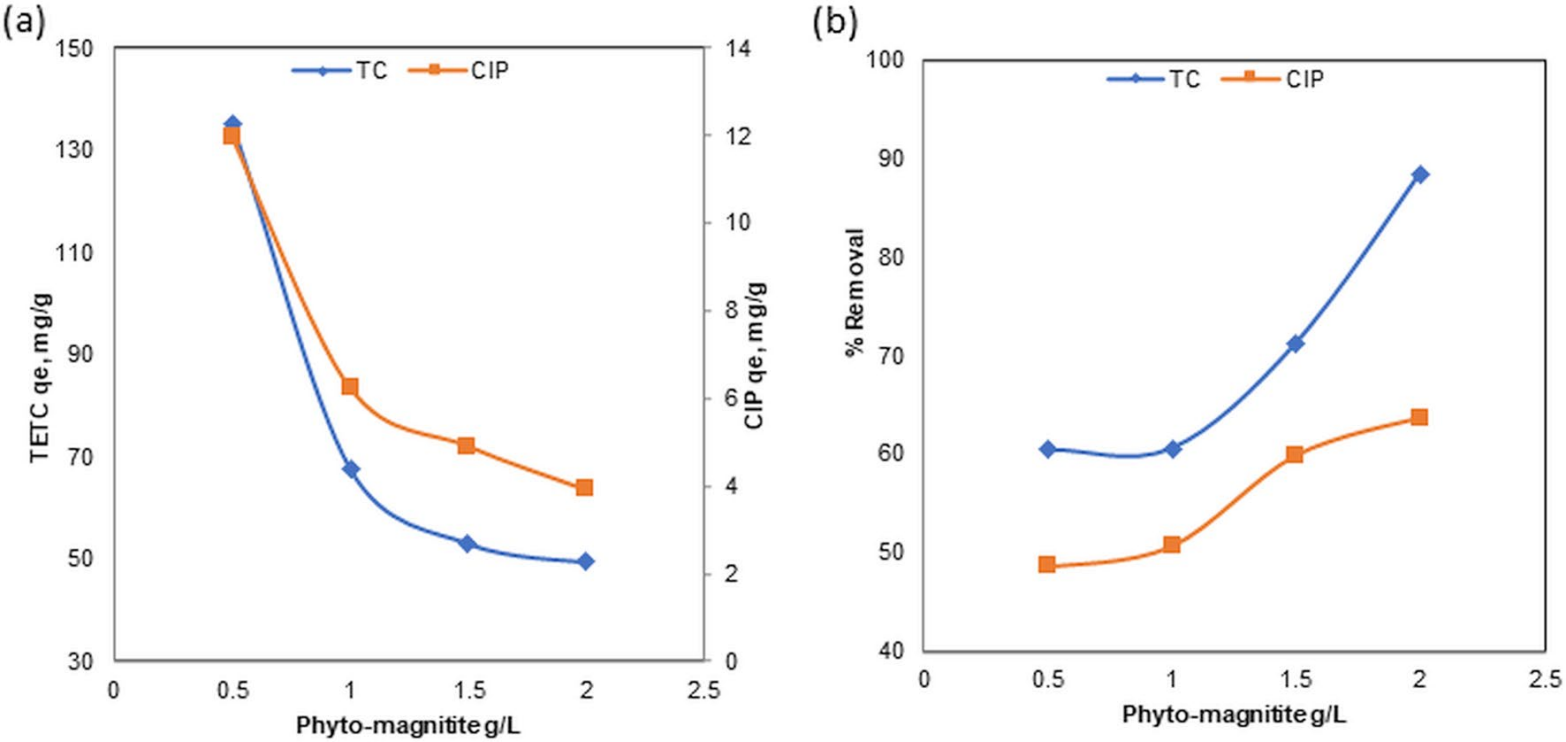
Effect of sorbent dose (**a**) adsorption capacity of Phyto-magnetite for TETC and CIP (**b**) removal efficiency of TETC and CIP.

The potential of the sorbent was examined by varying amounts of Phyto-magnetite at pH 7 and 25 °C in various initial TETC and CIP concentrations of 100 and 10 mg/L solutions. The sorbent doses were 0.5, 1, 1.5, and 2 g/L. At an initial TC concentration of 100 mg/L, the adsorption capacity was found to be 13, 5, 67.7, 53.5, and 49.4 mg/g, respectively (Fig. 7). The removal rate increased with increasing Phyto-magnetite dose, and the maximum removed efficiency achieved was 88% at 2 g/L of nanocomposite. Unlike TETC, CIP removal was not significant and adsorption capacity ranged between 1.7 and 11.3 mg/g while removal efficiency ranged between 48 and 63% only. The results indicated the ability of Phyto-magnetite to effectively remove and absorb high TETC even at a lower sorbent dose (60% at 0.5g/L). The results indicated also that Phyto-magnetite nanocomposite is not effective for removal of CIP even at higher doses.

**Figure 8:**
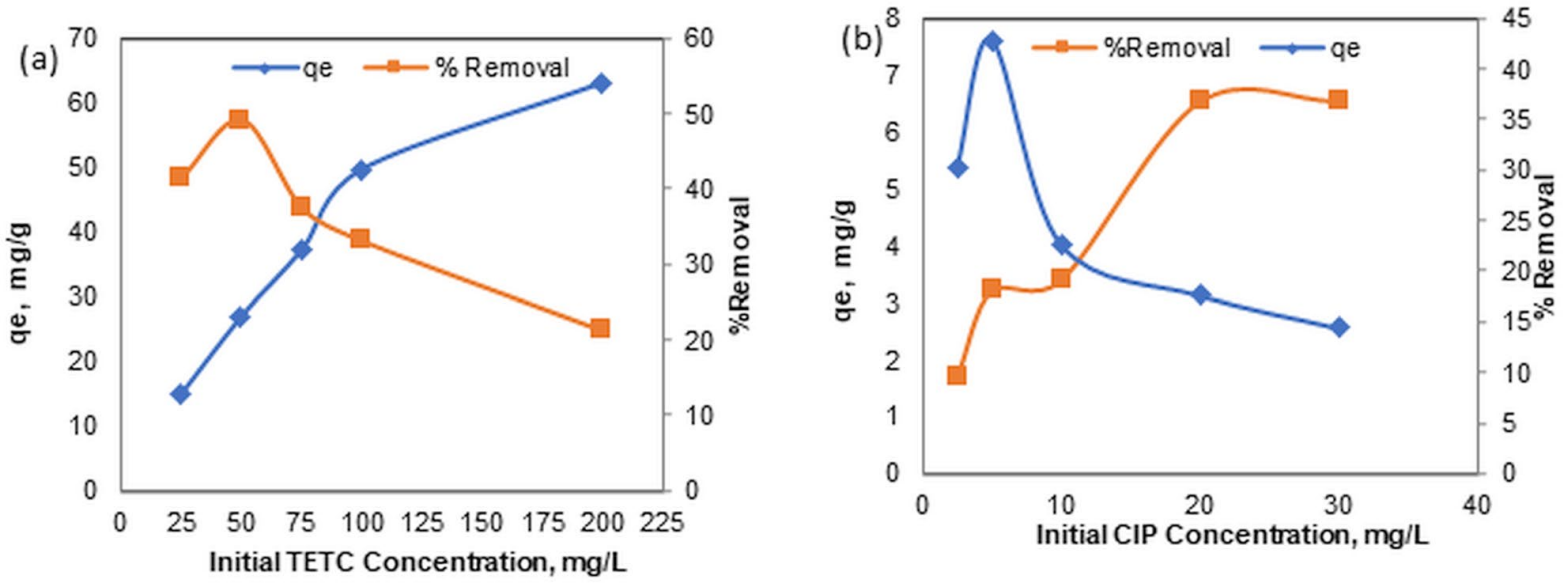
Effect of (**a**)TETC and (**b**) CIP different initial concentrations on adsorption capacity of Phyto -magnetite.

Figure 8 demonstrates the effects of varying the concentration of TETC (25–200 mg/L), assessed at a fixed amount of adsorbent (1g/L) and a pH level of 7. With an increase in TETC concentration from 25 mg/L to 200 mg/L, the removal efficiency correspondingly decreased from 53 to 21% as seen in Fig. 8a. The results revealed that accelerated adsorption occurred at lower TETC concentrations compared to higher concentrations. The adsorption data indicated that the adsorption capacity of Phyto-magnetite increased from 15.1 to 63 mg/g as a result of increasing the TETC concentration from 25 to 200 mg/L, due to the improved driving forces for mass transfer. The adsorption capacity of CIP and removal rate under different initial concentrations from 2.5 to 30 mg/L are presented in Fig. 8b. The results showed a poor adsorption capacity that ranged between 1.7 and 6.3 mg/g with a removal rate between 14 and 40% only.

#### Adsorption kinetics and isotherms

**Figure 9:**
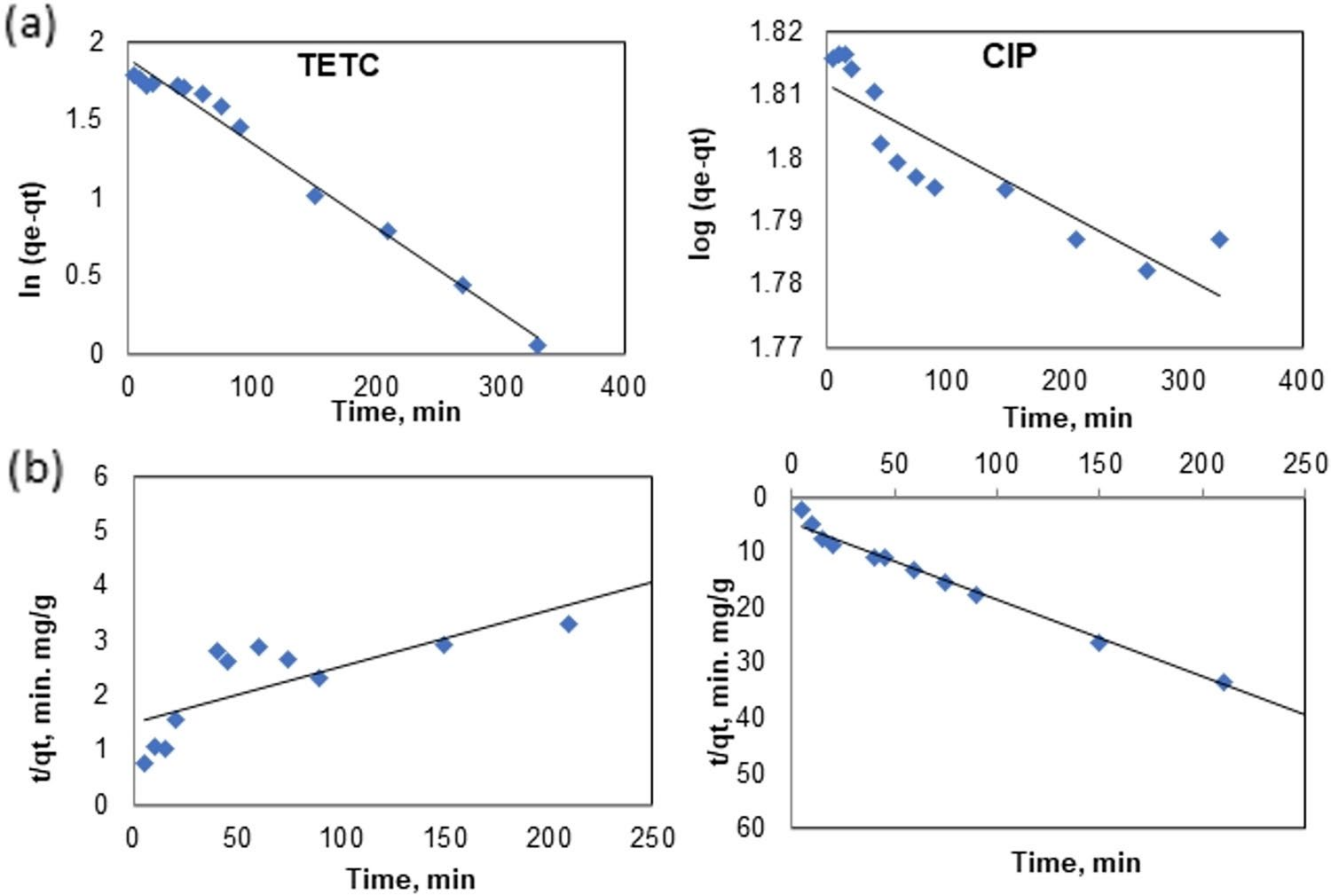
Adsorption kinetic models for TETC and CIP; (**a**) Pseudo-first-order, (**b**) Pseudo-second order.

Linear fits for the kinetic models of pseudo-first order, and pseudo-second order, are depicted in Fig. 9a,b, with the kinetic parameters tabulated in Table 2. Outcomes derived from these models suggest that the adsorption of TETC onto Phyto-magnetite adheres to a pseudo-first-order kinetic model. The R^2^ values obtained from this model (R^2^ > 0.98) were determined to be superior when juxtaposed with those observed in the pseudo-second order model (R^2^ = 0.808) as shown in Table 2. Moreover, the calculated values of (qe_cal_) were found to be in agreement with those of the experimental ones (qe_exp_). These findings for TETC antibiotics infer that the adsorption of TETC by Phyto-magnetite constitutes a physical adsorption process. On the contrary, kinetic studies demonstrated that CIP adsorption in Phyto-magnetite adhered to a pseudo-second order kinetic model, where R^2^ was 0.989 compared to 0.78 for the pseudo-first order model.

**Figure 10:**
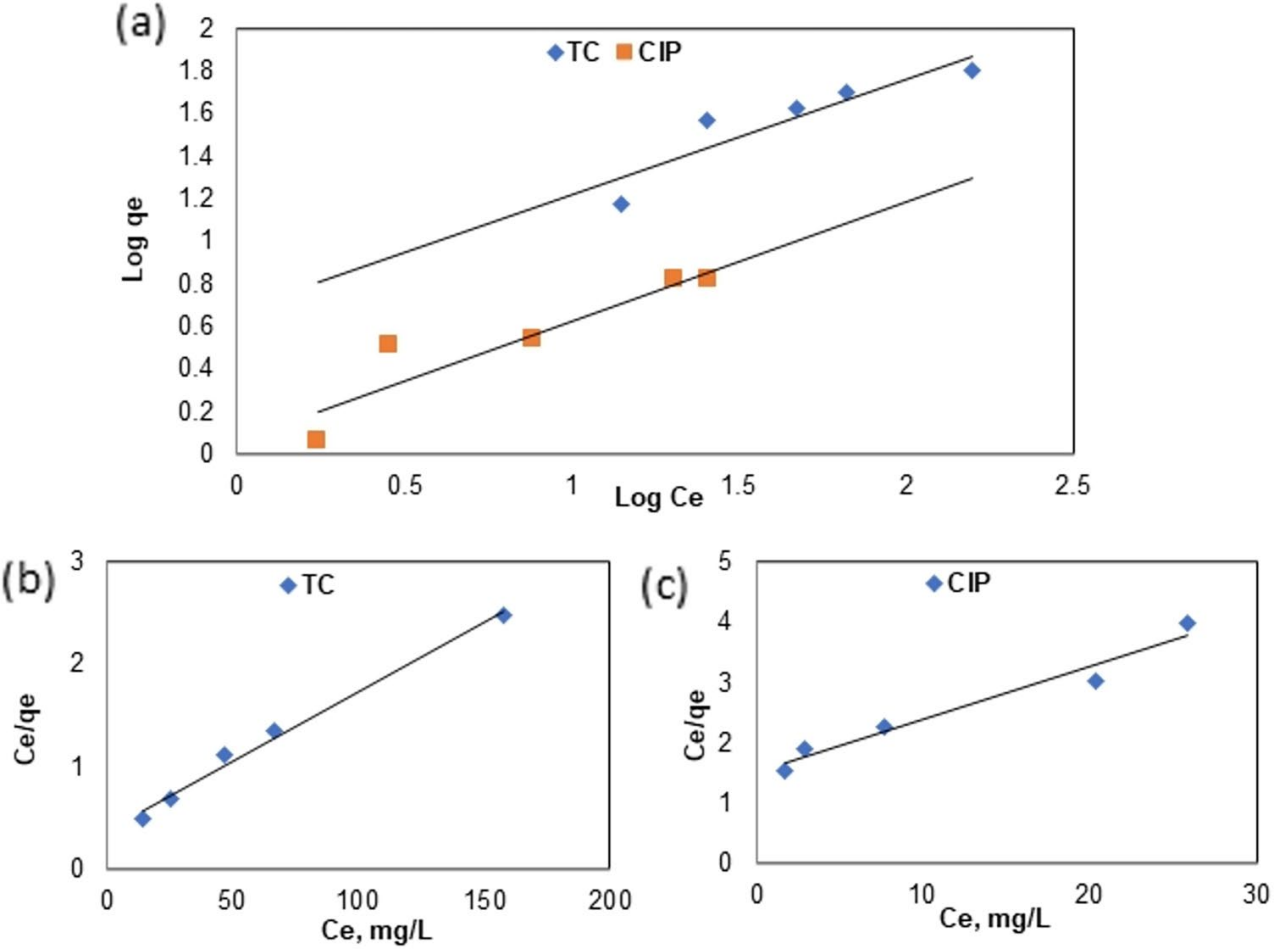
Adsorption isotherm models; (**a**) Langmuir, (**b**) Freundlich, for the adsorption of TETC and CIP onto Phyto-magnetite.

The adsorption of TETC and CIP by Phyto-magnetite was analyzed using Freundlich and Langmuir isotherm models (Table 2), and the fit quality was evaluated based on the correlation coefficient. Upon comparing the R^2^ values of these models (Table 2, Fig. 10a–c), it was determined that the Langmuir model had lower correlation coefficients (0.83 and 0.85) than the Freundlich model (0.99 and 0.95) for both TETC and CIP. Consequently, it was deduced that the process adheres to the Freundlich isotherm. Furthermore, the maximum adsorption capacity (qm) was calculated from the Langmuir first-order. The linear plot for Freundlich adsorption isotherm (Fig. 10b,c) suggests that TETC's adsorption onto Phyto-magnetite occurs via physical adsorption.

## Discussion

### Evaluation of main physico-chemical parameters

As shown in Fig. 2a, SP4 (El Nazla canal) recorded the highest TDS values in all seasons, indicating that it may contain a source of pollution, however, the readings did not exceed the national regulatory limit (500 mg/L). Through investigation of this canal, it was found that it is slightly contaminated with agriculture drainage water in small amounts. The data show an increase in TDS values in recent years compared to previous data published on evaluations of water quality near the same sites. For example, Waheed et al.^33^ reported TDS values between 255 and 280 mg/L for sites near SP1, SP2, SP3, and SP6. The presence of excessive TDS can also lead to the accumulation of harmful ions, affecting plant health and growth. BOD and COD are critical parameter used to assess the organic pollution load in water bodies. During summer, higher values of COD and BOD were observed. These higher values during summer can be attributed to increased organic matter and nutrient runoff from agricultural activities, requiring increased attention to water treatment and nutrient management during this period. The observed BOD level slightly exceeded the acceptable limits, suggesting that irrigation water is not highly polluted with organic matter. However, it emphasizes the importance of responsible irrigation practices to minimize the introduction of pollutants into water sources.

In group B, showed a high concentration of TDS values than in the summer months and all the samples. This was attributed to the large amount of drainage water and sewage from some households discharged directly to these canals without treatment. The water in these canals is affected by waste discharges from household activities, wastewater from WWTPs and agricultural drainage water, as well as the lower water volume, which maximizes the concentration of dissolved salt. This explanation is consistent with^34^.Unfortunately, these canals are used by local farmers for irrigation and these high concentrations of TDS can adversely affect soil structure and reduce crop yields, necessitating proper irrigation scheduling and drainage practices.

The findings reveal that the levels of chemical oxygen demand (COD) and biochemical oxygen demand (BOD) increase significantly during the summer months due to the higher water consumption and increased discharge of sewage into the canal system. This heightened organic load causing elevated COD and BOD levels is further exacerbated by agricultural activities, including the disposal of plant and animal waste from irrigation systems. This collective increase in COD and BOD levels during the summer season has been similarly documented in a study conducted in Korea^24^. Moreover, the surge in domestic usage and human activities during the summer season notably contributes to the rise in wastewater effluent from wastewater treatment plants (WWTPs) in the Fayoum region, which is then released into various waterways within the area. Therefore, these findings can be implemented to guide environmental protection and mitigation efforts in the specific case study region to address issues of water pollution and sewage discharge. Furthermore, it is essential to consider the long-term implications and consequences of such heightened levels of COD and BOD during the summer months. This increase in organic load not only poses a threat to the aquatic ecosystem but also has significant impacts on water quality and public health^35^. The excessive discharge of sewage into the canal system can lead to the contamination of drinking water sources and recreational areas, making them unfit for human use. This, in turn, may result in the outbreak of waterborne diseases and other adverse health effects. To tackle these issues effectively, a multi-faceted approach is required. Firstly, it is crucial to regulate and monitor the discharge of sewage from both domestic and agricultural sources. Implementing stricter regulations and ensuring proper wastewater management practices can help minimize the release of harmful pollutants into the waterways. Additionally, promoting the use of advanced treatment technologies in wastewater treatment plants can significantly reduce the levels of COD and BOD in the effluent^35^. Furthermore, raising awareness among the local community about the importance of water conservation and responsible water usage can play a pivotal role in preventing excessive water consumption. Encouraging sustainable agricultural practices that prioritize efficient irrigation methods and proper waste disposal can also contribute to reducing the organic load in the canal system. Moreover, it is imperative to establish effective collaborations between government agencies, environmental organizations, and local communities to address the issue comprehensively. This can include joint efforts in conducting research, developing and implementing strategies, and providing necessary resources to combat water pollution and sewage discharge effectively^36^. By taking these measures and utilizing the findings from this study, the specific case study region can take proactive steps towards environmental protection and mitigation efforts. These actions will not only safeguard the local water resources but also ensure the well-being and sustainable development of the community in the long run.

Group C showed no great variation in the TDS values during different summer and winter seasons, which could be explained as these drains always receive water from various sources and the water levels in these drains are constant. The results in Fig. 2c showed that SP13 recorded the highest TDS values (about 6000 mg/L). The high concentration of salts in the drains may be attributed to these drains receiving massive quantities of untreated sewage, wastewater from fish farms and drainage water. However, the concentration in SP13 also showed high concentrations of anions and cations, while other samples were similar. The increase in contraction during winter can be attributed to the decrease in water levels in the canal during winter. The composition of the TDS concentration may be the formation of Na–SO_4_ and Na–Cl, Ca–Mg–Cl–SO_4_. However, the analysis shows that the concentration of SO_4_^2−^ ions is higher than that of Cl ions (Fig. 4S–5S), indicating that the source of SO_4_^2−^ is sewage contamination. The concentration of Na ions in all collected samples is very high compared to Ca^2+^, Mg^2+^, and K^+^, suggesting that the source of the increase in Na in polluted canals may be water drainage. The relative increase in SO_4_^2−^ and Cl^−^, as well as the relative increase in Na relative to Ca^2+^ and Mg^2+^, indicate the presence of evaporators in the area studied. As Fayoum is always dry and hot, Na^+^, Mg^2+^, Cl, and SO_4_^2−^ of dissolved Na ^+^, Cl^−^, and SO_4_^2−^ concentrations continue to accumulate in channels dominated by Na and Cl, while Ca^2+^ and Mg^2+^ precipitation forms carbonates^33^. The results in Supplementary Fig. 4S and 5S confirm this, showing a higher concentration of Na, Cl, and sulfur in summer than in winter.

### Microbiological characterization

The results of bacteriological analysis serve as crucial indicators for evaluating water quality in many aspects. The presence of total coliform bacteria (TC) in potable water has the potential to induce severe illnesses in humans; however, their primary function is to estimate the likelihood of the existence of other more pathogenic microorganisms linked to wastewater. The detection of TC bacteria suggests the plausible presence of fecal matter and bacteria responsible for causing diseases^37^. Fecal coliforms (FC) and fecal streptococci (FS) are bacterial species that, when present, signal potential contamination of water sources by human or animal waste^38^. It is apparent that the FC and FS values are significantly increased in the summer season compared to the winter season. The bacterial counts founds in these canals are consisted with other studies carried out on other Egyptian water canals^39,40^. In the study by El-Meihy^39^, the mean counts of TC were ranged between 5.1 × 10^3^ and 2.7 × 10^5^ CFU/100 mL in water samples collected from Rosetta branch during winter and summer, while in Damietta Branch waters were ranged between 8 × 10^3^ and 5.7 × 10^4^ /100mL water for TC. This variation can be attributed to increased domestic and agricultural activities during the warmer months^41^ posited that fecal bacterial growth in surface water is facilitated by nutrient sources stemming from household and agricultural waste. The SP11 water sampling site showed the highest FS concentration, as it was located close to the outlet of the household pipes. The presence of discarded waste materials and deceased animals in drains and irrigation canals further contributes to elevated contamination levels among varying bacterial species. Such scenarios pose significant health risks, particularly when untreated water is used for drinking purposes. A report byIOB^42^ highlighted that exposure to contaminated water sources contributes to an increased prevalence of waterborne diseases within Fayoum watershed. Risks include direct exposure by standing or swimming in contaminated waters, as demonstrated by numerous observations of local children participating in such activities. In addition, indirect infection can occur through the transmission of pathogens to crops and produce, posing a potential health threat. It is important to note that the presence of total coliforms, fecal coliforms, and algae can indicate potential sources of contamination and environmental stressors in the water. Regular monitoring of water quality, coupled with appropriate water treatment and nutrient management practices, are critical to ensure safe and sustainable irrigation water throughout the year.

The abundance of fungi in the canal water during the summer in the Fayoum region is mainly due to the significantly large amount of organic matter and nutrients. These substances, such as dead plants, decaying vegetation, and various forms of algae, provide a plentiful source for fungal organisms, facilitating their growth and development^43^. Moreover, these factors contribute to the creation of an optimal environment that supports the thriving and proliferation of diverse fungal life forms. Furthermore, the stability of the water temperature during the summer months plays a key role in promoting the accelerated growth and heightened activity of fungi. This consistent temperature, combined with the presence of abundant resources, facilitates the efficient utilization of organic matter and nutrients by the fungal population in the canal water^44^. As a result, the fungal community experiences a significant boost in their reproductive capacities, leading to the emergence of a diverse array of fungal species within the ecosystem. It is important to note that not only do these fungal populations flourish during the summer months, but their levels also exhibit notable fluctuations throughout the seasons, underscoring the dynamic and ever-evolving nature of microbial communities within aquatic ecosystems. These fluctuations can be attributed to various factors, including changes in nutrient availability, water flow dynamics, and interactions with other microorganisms^45^. Understanding and monitoring these patterns is of utmost importance in order to effectively implement successful management strategies aimed at safeguarding and preserving water quality and the overall public health in the region^46^. By comprehending the intricacies of these fungal population dynamics, authorities can develop and enforce targeted measures to ensure the long-term integrity of the canal water. This may involve implementing measures to mitigate excessive nutrient inputs, improving water treatment processes, and enhancing microbial diversity within the ecosystem. Furthermore, promoting public awareness and education about the importance of maintaining a balanced and healthy aquatic ecosystem can also contribute to the preservation of water quality. Overall, the presence and behavior of fungi in the canal water during the summer months are influenced by a multitude of factors, including the availability of organic matter and nutrients, favorable temperature conditions, and interactions with other microorganisms. By recognizing the complex nature of these interactions and adopting proactive management approaches, it is possible to maintain a harmonious balance in the canal water, ensuring its long-term sustainability and the well-being of the surrounding ecosystems and communities.

### Antibiotic analysis

The observed elevation in antibiotic usage during the winter months may be attributed to the increased prevalence of seasonal illnesses, such as common colds, respiratory tract infections, influenza, and other climate-related conditions. The lower concentrations detected in the summer season could potentially result from heightened water consumption leading to the dilution of antibiotic concentrations within the effluent of wastewater treatment plants. Additionally, it is plausible that the escalated degradation of detected antibiotics occurs due to increased exposure to ultraviolet radiation from sunlight, which is more prevalent during the summer months. These findings corroborate numerous studies that have reported elevated pharmaceutical concentrations in winter in comparison to summer season levels^47–51^. While these concentrations might seem relatively low, the continuous use of antibiotics in human and animal healthcare can result in their persistence and accumulation in water bodies over time. Antibiotic-resistant bacteria can compromise the effectiveness of antibiotics in treating infections, making it critical to address this issue to preserve the efficacy of these medications.

### Adsorption of antibiotics on Phyto-magnetite

The results of this study revolve around the evaluation of a Phyto-magnetite nanocomposite's effectiveness in the removal of two common antibiotics, tetracycline (TETC) and ciprofloxacin (CIP), from wastewater. These findings are significant in the context of wastewater treatment and environmental remediation, as antibiotics in wastewater pose a considerable concern due to their potential impact on aquatic ecosystems and the development of antibiotic-resistant bacteria. The results revealed intriguing insights into the kinetics of the adsorption process. Both TETC and CIP initially displayed rapid adsorption, with removal rates increasing significantly. This phase was most pronounced during the first 200 min for TETC, during which over 70% of TETC was removed. After this point, the adsorption rate began to slow, reaching equilibrium around 240 min. In the case of CIP, there was an initial increase in removal during the first 90 min, but after 150 min, the adsorption rate began to decline. Notably, the optimum contact time for TETC removal was established at 300 min, while the best timeframe for CIP adsorption differed, underscoring differences in their adsorption mechanisms.

At an initial TETC concentration of 100 mg/L, increasing the dosage of Phyto-magnetite led to enhanced adsorption capacity, with a maximum removal efficiency of 88% achieved at 2 g/L of the nanocomposite. In stark contrast, CIP removal remained modest even at higher sorbent doses. These results emphasize the potential of Phyto-magnetite for efficient TETC removal, even at lower sorbent doses, while CIP exhibited limited removal efficiency. As the initial TETC concentration increased from 25 to 200 mg/L, the removal efficiency decreased, highlighting competitive adsorption mechanisms at higher concentrations. This phenomenon can be attributed to competitive adsorption mechanisms at elevated concentrations^52,53^. However, the adsorption capacity increased with increasing TETC concentration, underscoring the role of driving forces in mass transfer^54^.This phenomenon was observed to be more efficient than a previous study by Abdelfatah et al.^32^ who obtained maximum adsorptipn capacity of 51.5 mg/g at initial ion concentration of 200 mg/L. Conversely, CIP displayed low adsorption capacity and removal rates across different initial concentrations. The results obtained are better than those obtained by the study employed kinetic models, including pseudo-first order and pseudo-second order, to elucidate the adsorption mechanisms. For TETC, the results suggested that the adsorption process adheres to a pseudo-first-order kinetic model, indicating a physical adsorption mechanism. In contrast, CIP adsorption followed a pseudo-second-order kinetic model, suggesting a different adsorption process. These findings shed light on the different kinetics governing TETC and CIP adsorption onto Phyto-magnetite. The adsorption isotherms, which describe the relationship between adsorbate concentration and the amount adsorbed at equilibrium, were also examined. Both TETC and CIP adsorption data were analyzed using Freundlich and Langmuir isotherm models. The Freundlich model provided a better fit to the data for both antibiotics compared to the Langmuir model, suggesting a heterogeneous, multilayer adsorption mechanism. The Freundlich model exhibited high correlation coefficients, further supporting its suitability for describing the adsorption process. In summary, the results underscore the potential of the synthesized Phyto-magnetite nanocomposite for efficient tetracycline removal from wastewater, particularly at lower sorbent doses and higher initial concentrations. The data also offer insights into the adsorption kinetics and isotherms, which suggest distinct mechanisms for tetracycline and ciprofloxacin adsorption. This research contributes to the body of knowledge concerning the use of nanocomposite materials for the removal of pharmaceutical contaminants from wastewater, which is of paramount importance for environmental and public health.

## Conclusions

This study presents a novel physicochemical characterization of water sources in Fayoum, Egypt, providing critical insights into the suitability of water for agricultural purposes and identifying potential environmental risks. The key findings indicate that irrigation water generally meets acceptable limits for essential parameters such as pH, electrical conductivity (EC), and total dissolved solids (TDS), making it suitable for irrigation during both the winter and summer seasons. However, the study also uncovered significant issues with high contamination levels in irrigation canals due to the mixing with drains or the discharge of untreated sewage. Microbial indicators, including total coliforms and fecal coliforms, exhibited seasonal fluctuations, underscoring the need for continuous water quality monitoring and proper sanitation practices to ensure water safety and public health protection. The seasonal patterns in algal concentrations highlight the importance of managing nutrient inputs to prevent excessive algal growth and maintain water quality.

A critical environmental concern identified is the presence of antibiotics in irrigation water, which could lead to the development of antibiotic-resistant bacteria and potential waterborne diseases. Notably, the application of Phyto-magnetite as an adsorbent demonstrated significant potential in mitigating water pollution. The adsorption process effectively reduced antibiotic concentrations to permissible levels, indicating that Phyto-magnetite is a viable and eco-friendly alternative for water treatment. Despite these promising findings, further research is necessary to fully understand the adsorption mechanism of Phyto-magnetite and to optimize the process to ensure its economic feasibility and scalability. Future studies should focus on enhancing the Phyto-magnetite treatment method, exploring additional contaminants, and investigating long-term environmental impacts. This research sets the stage for developing sustainable water management strategies and improving water quality in agricultural settings, ultimately contributing to environmental protection and public health.

### Supplementary Information

Supplementary Information.

## Data Availability

All authors ensure that all data and materials and software applications or custom code support their published claims and comply with domain standards. The datasets used or analyzed during the current study are available from the corresponding author on reasonable request.
