# Supplementary material for: A comprehensive assessment of water quality in Fayoum depression, Egypt: identifying contaminants, antibiotic pollution, and adsorption treatability study for remediation
Source: Sci Rep. 2024 Aug 14;14:18849. doi: 10.1038/s41598-024-68990-8 (PMC11324722; doi:10.1038/s41598-024-68990-8)
Supplement: Supplementary file 1 — Supplementary Information. [file 41598_2024_68990_MOESM1_ESM.pdf]

Supplementary materials for

A Comprehensive Assessment of Water Quality in Fayoum Depression, Egypt: Identifying Contaminants, Antibiotic Pollution, and Adsorption Treatability Study for Remediation

A Comprehensive Assessment of Water Quality in Fayoum Depression, Egypt: Identifying Contaminants, Antibiotic Pollution, and Adsorption Treatability Study for Remediation

Mai Sayed Fouad [1], Emad Fawzy Mustafa[2], Mohamed Saad Hellal[3*], Mai Ali Mwaheb[1*]

[1]Botany department, Faculty of Science, Fayoum University, Fayoum 63514, Egypt

[2]Water Management Research Institute, National Water Research Center NWRC, Egypt

[3]Water Pollution Research Department, National Research Centre, Cairo 12622, Egypt.

*Correspondence: Mohamed Saad Hellal, mohammed_saadh@yahoo.com. Water Pollution Research Department, National Research Centre, Cairo 12622, Egypt.

*Correspondence: Mai Ali Mwaheb mam08@fayoum.edu.eg, Botany department, Faculty of Science, Fayoum University, Fayoum 63514, Egypt.

Method of Phyto-magnetite nanocomposite preparation

Phragmites australis plant specimens were collected from Fayoum depression canals, located in Egypt. Specimens were separated into rhizomes before being washed thrice with deionized water to eliminate contaminants and debris, then left to air dry. Plant materials (rhizomes) were subsequently cut into small fragments, oven-dried at 60°C until thoroughly desiccated, then ground into fine powders. Aqueous extracts of rhizomes were prepared by adding ten grams of each organ to 200 mL of distilled water, heating, and stirring at 500 rpm with a magnetic stirrer (Thermo-fisher, USA) until boiling for approximately 20 minutes. Hot solutions were filtered through Whatman filter paper (Fig. 1S). Phyto-magnetite nanoparticles was prepared according to Jin et al[1] by dissolving 10.8g sample of $FeCl_3 \cdot 6H_2O$ and 4 g of $FeCl_2 \cdot 4H_2O$ were in 60 mL mixture solution containing 90% deionized water and 10% *Phragmites australis* extract to prepare a stock solution then 1.7 mL of HCl was added to the solution. The stock solution was then added drop by drop to 500 mL of 1.5 mol/L NaOH under vigorous stirring using a non-magnetic stirrer at 80°C. The Phyto-magnetite nanoparticles were precipitated and then washed several times with deionized water and ethanol. The Fe_3O_4 nanoparticles were then

resuspended in deionized water. The as-prepared Fe_3O_4 nanoparticles were stored under bench-top conditions until.

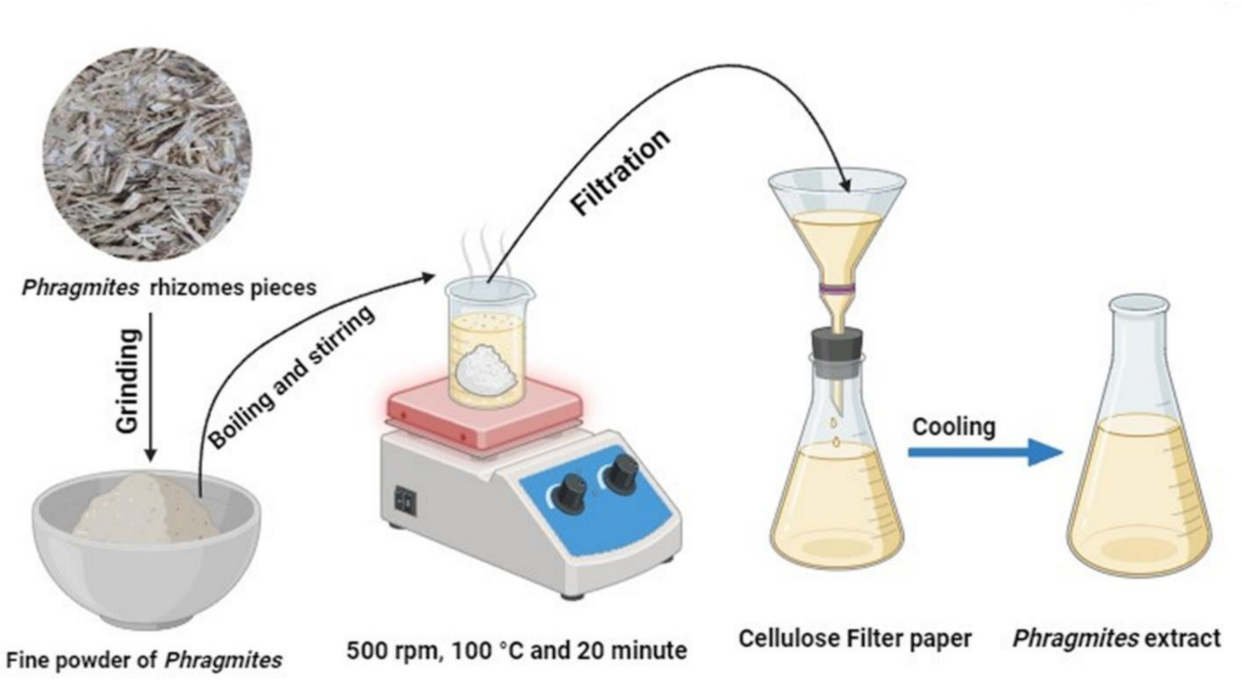

Fig. 1S Schematilc illustaration of extraction

Characterization of Phyto-magnetite nanoparticle

The physico-chemical characterization of Phyto-magnetite was carried out in terms of Transmission electron microscopy (TEM), surface area, X-ray diffraction (XRD) and FTIR spectrometry. TEM micrographs were performed on JEOL JEM-2100 high resolution transmission electron microscope at an accelerating voltage of 200 kV, respectively. Samples for TEM were prepared by placing a droplet of colloid suspension in respective solvent on a Formvar carbon-coated, 300-mesh copper grid (Ted Pella) and allowing them to evaporate in air at ambient conditions. Fig. 2S shows TEM micrographes of the phyto-magntite nano-composite masses resemble structures in that they include

spherical, uniform, and virtually regular pores and voids with diameters ranging from 3.5 to 4.9 nm. Selected area electron diffraction (SAED) showed a concentric circle with spots of diffraction scattered around the edges of a particular region.

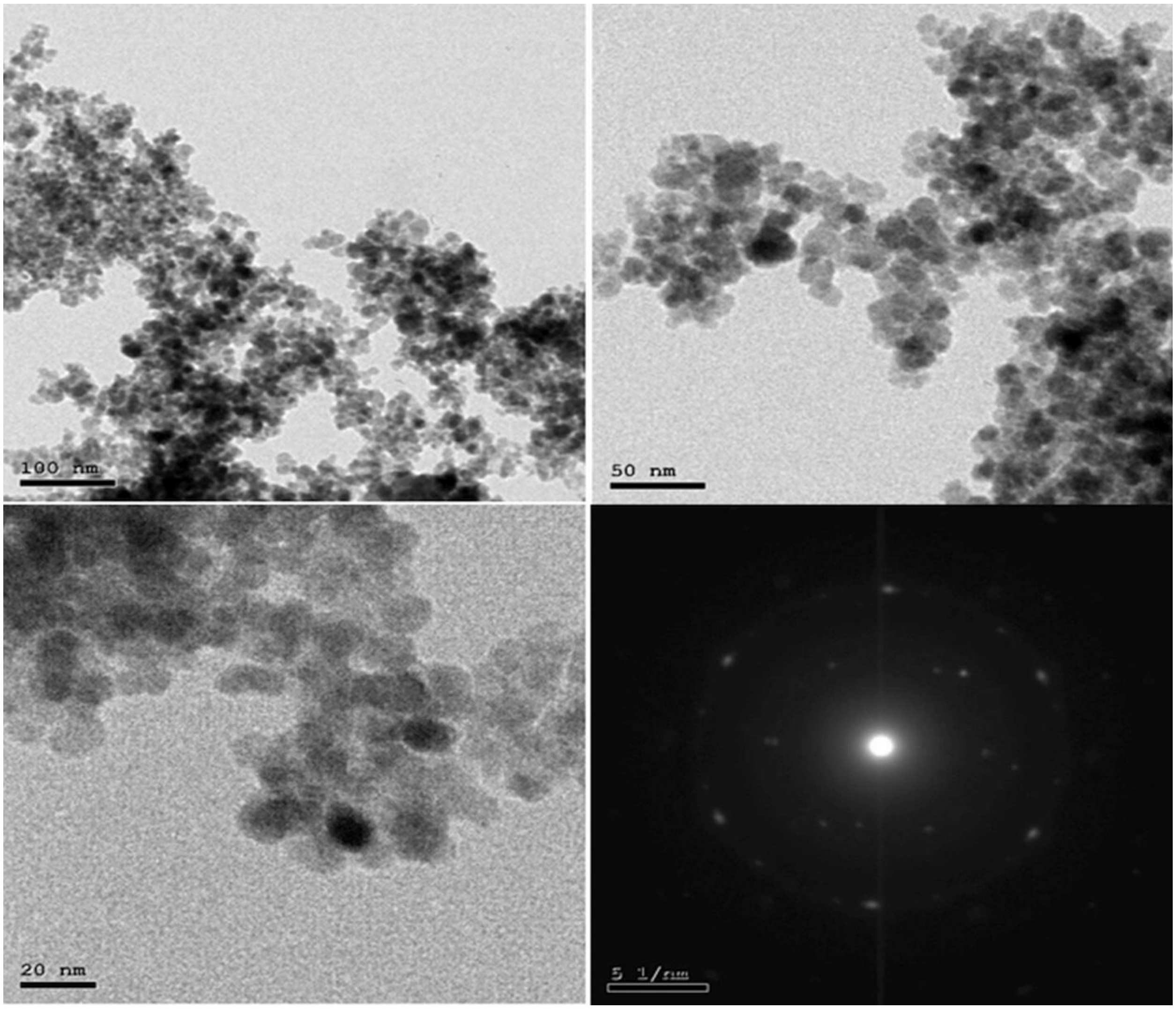

Fig. 2S TEM images of magnetite at different magnefications and SAED patteren

XRD pattern has been performed using XPERT-PRO Powder Diffractometer system, with 2 theta (10° - 80°), with Minimum step size 2Theta: 0.001, and at wavelength (Kα) = 1.54614°. The crystalline structures of the as-prepared material Fe3O_4 Nanomaterial were performed by XRD (Fig. 3S-a). Fe_3O_4 structure displays nine characteristic peaks at 2θ = 18.4◦, 30.28◦, 35.67◦, 37.32◦, 43.36◦, 47.48◦, 53.80◦, 57.36◦ and 63.00◦ and 74.55◦

which corresponds to the (111), (220), (311), (222), (400), (331) (422), (511) and (440) and (533) planes of cubic Fe_3O_4 Reference code: 01-075-0449, respectively. FTIR spectroscopy was carried out using FT-IR vertex 70 RAM II, Bruker Spectrometer. FTIR spectra of is shown in (Fig. 3S-b) that shows the characteristic peak of iron oxides, i.e., Fe-O at 543 cm^{-1} . The literature reports this peak at 548 cm^{-1} [2,3]. Also, the spectrum exhibits modes typical for organic groups in regions of 860‐1650 cm^{-1}.

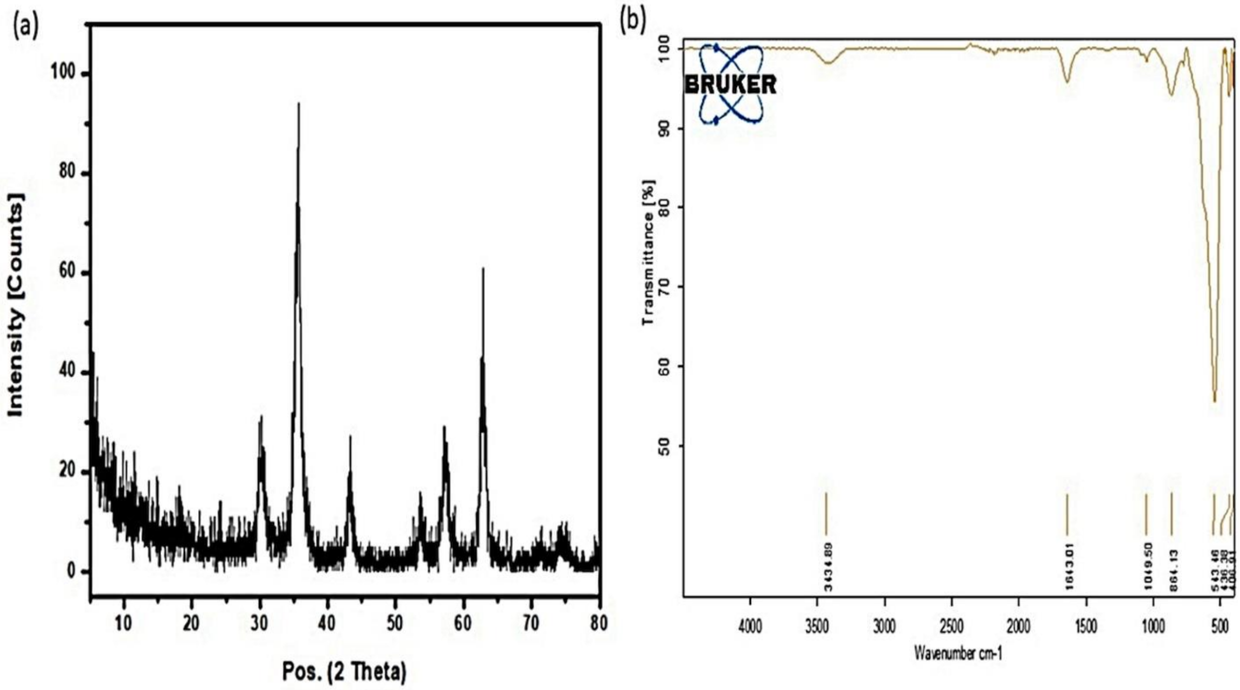

Fig. 3S (a), XRD pattern and (b), FTIR of the prepared nanoparticles

A surface area characteristic was carried out using Quantachrome (USA; Nova 2000 series) for N_2 physisorption isotherm studies. Due to the presence of interparticle holes between the NPs, adsorption and desorption analysis of the fabricated Si NPs revealed a superior Brunauer–Emmett Teller (BET) surface area (186.5 m^2/g), Barrett-Joyner-Halenda (BJH) surface area (129.7 m^2/g), a total pore volume (0.54 cm^3/g), and an average particle radius (1.92 nm).

Adsorption Study calculations

The adsorbed amount of antibiotic (q_e) onto the developed nanocomposite adsorbent was calculated as shown in equation (1).

$$q_e \text{ (mg /g)} = \frac{(Ci - Cf)V}{m} \qquad (1)$$

Where q_e (mg/g) is the number of pollutants adsorbed per unit weight of the adsorbent at equilibrium. C_i and C_f are the initial and final nickel ion concentration. While V is the volume of the aqueous solution and m (g) represent the weight of the nano adsorbent.

Kinetic modelling and adsorption isotherms

Pseudo first order (PFO), and pseudo second order (PSO), models were used to analyze the adsorption processes. The linear form of the PFO, and PSO models were as follows (Eq. 2-4),

Pseudo first order: $$\ln(q_e - q_t) = \ln q_e - k_{PFO}t \qquad (2)$$

Pseudo Second order: $$\frac{t}{q_t} = \frac{1}{k_{PSO} \times q_e^2} + \frac{1}{q_e} \qquad (3)$$

Where q_t and q_e are connected to the quantity of TC and CIP adsorbed on per unit weight of Phyto-magnetite at time 't' and equilibrium, respectively. The constants K_{PFO} and K_{PSO} are in PFO, and PSO respectively. K_{PFO} and $K_{PSO\ D}$ are estimated through the slope and intercept of the curves of plot of In(q_e-q_t) versus 't', t/q_t versus 't' .The capacity of Phyto-magnetite on TC and CIP sequestration was examined using isotherm models i.e. Freundlich and Langmuir; the linear forms of the models are explained as follows (eq. 4-5).

Freundlich isotherm: $$\log q_e = \log K_F + \frac{1}{n}\log C_e \qquad (4)$$

Where, C_e and q_e are the equilibrium concentration (mg/L) and equilibrium adsorption capacity (mg/g) of antibiotics, respectively. From the slope and intercept of log (q_e) versus log (C_e), the Freundlich parameter values, 1/n and KF, were calculated.

Langmuir isotherm: $$\frac{C_e}{q_e} = \frac{1}{q_m \times K_L} + \frac{C_e}{q_m} \qquad (5)$$

Where q_m=maximum adsorption capacity of nanocomposite; K_L= Langmuir equilibrium constant (L /mg). The Langmuir isotherm assumes that the adsorption of TC and CIP on the surface of Phyto-magnetite in the monolayer. The values of q_m and K_L were estimated through the slope and intercept of plots of $1/q_e$ versus $1/C_e$.

Table 1S Regulations limits of chemical and biological parameters in irrigation canals and drains.

No.	Parameters	Unit	Irrigation Canal	Drainage canal
**1**	pH	---	6.5-8.5	6-9
**2**	DO	mg/l	6	--
**3**	TDS	mg/l	500	1000
**4**	NH_4	mg/l	0.5	--
**5**	NO_3	mg/l	2	--
**6**	BOD	mg/l	6	30
**7**	COD	mg/l	10	50
**8**	Cd	mg/l	0.001	0.03
**9**	Cu	mg/l	0.01	1
**10**	Fe	mg/l	0.5	3
**11**	Mn	mg/l	0.2	2
**12**	Zn	mg/l	0.01	2
**13**	Pb	mg/l	0.01	0.1
**14**	Ni	mg/l	0.02	0.1
**15**	Total Coliform	MPN/100 mL	--	1000

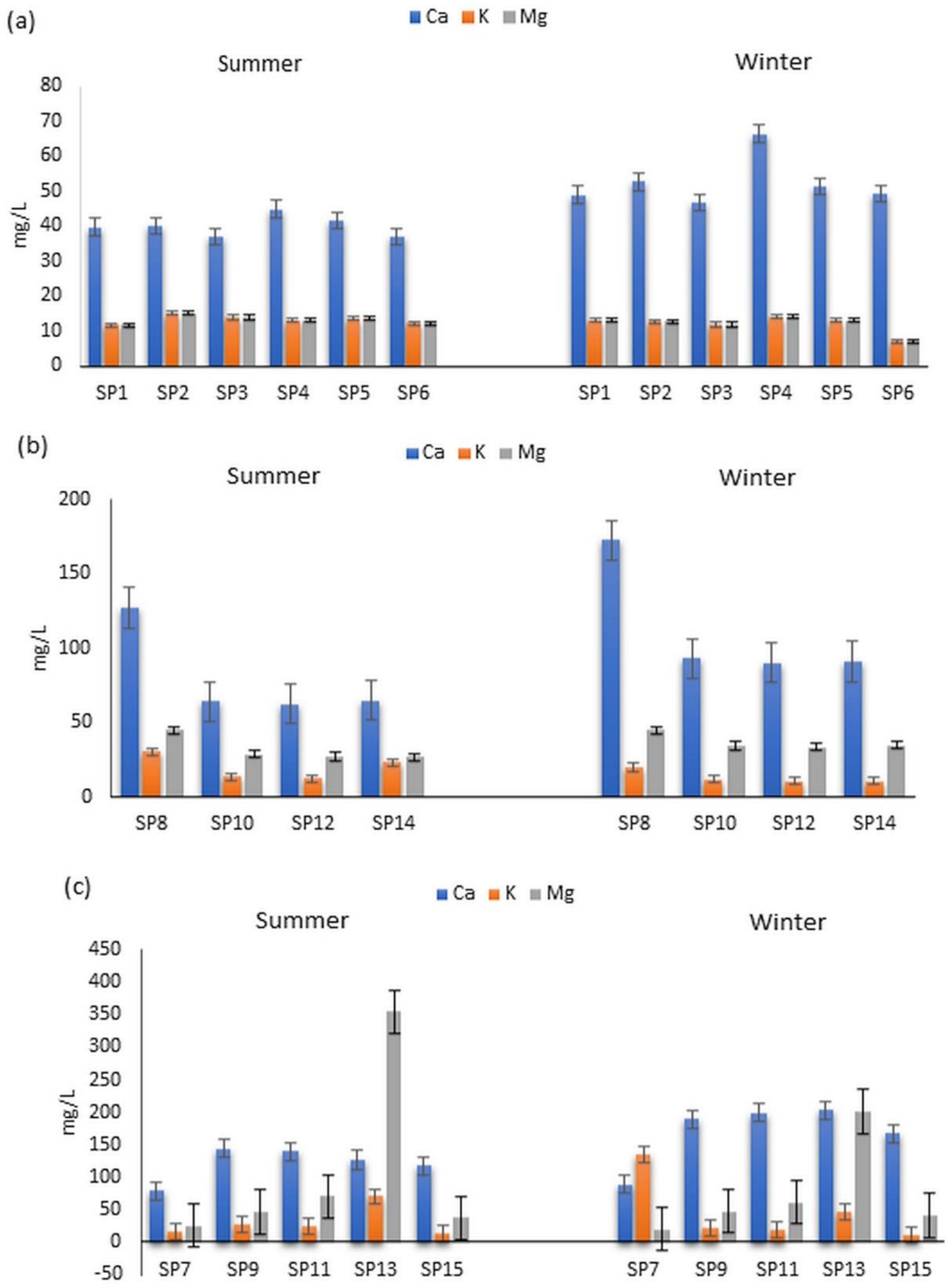

Fig. 4S Ca, Mg and K average concentration in different sampling sites (Group A, B, C) during summer and winter.

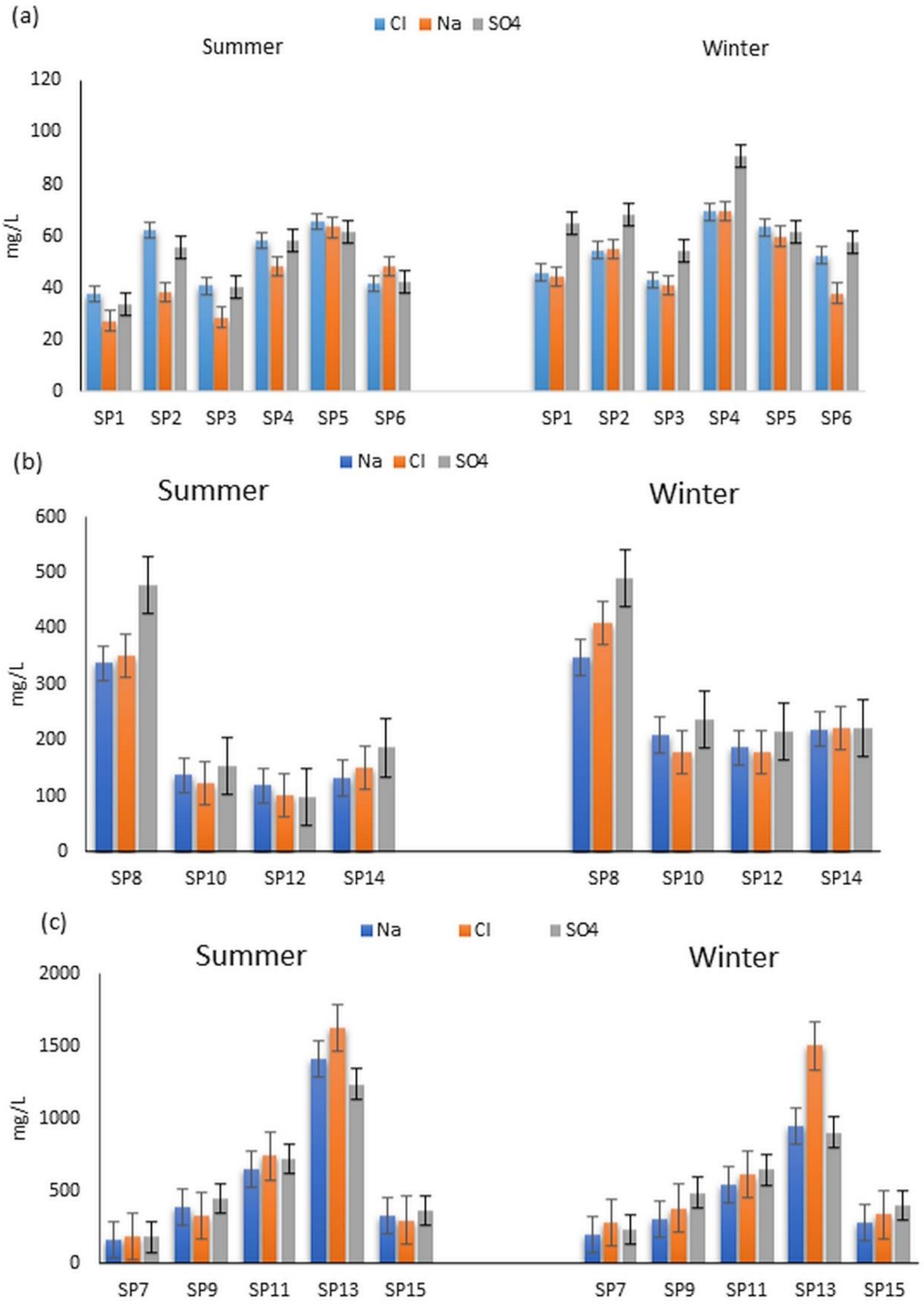

Fig. 5S Na, Cl and SO4 average concentration in different waster sampling sites (Group A, B, C) during summer and winter.

Table 2S Results of physiochemical and metals parameters of the water sample sites in Group A (SP1-SP6) during summer and winter.

Parameters	Unit	Summer				Winter			
		Min	Max	Mean	SD	Min	Max	Mean	SD
pH	--	7.20	9.75	7.81	0.47	7.40	7.99	7.73	0.19
Total Alkalinity	mg/L	135	243	188.59	27.25	133	240	190.21	21.72
EC	μS/cm	431.67	786.67	578.27	91.55	363.33	791.67	615.96	115.58
TDS	mg/L	259	472	346.96	54.93	218	475	369.57	69.35
NH3	mg/L	0.10	16.	1.39	2.96	0.01	22	4.06	7.16
BOD	mg/L	2.0	10.1	9.62	4.7	2	14	8.57	2.14
COD	mg/L	4.0	17.4	11.01	6.8	6	19.3	4.27	13.62
Ca	mg/L	30.58	62.39	41.06	8.63	44	69	52.57	6.89
K	mg/L	5	15	7.64	2.12	5	9	6.57	1.19
Mg	mg/L	9.10	18.40	13.36	2.18	6.85	18.40	12.08	3.10
Na	mg/L	30	75	51.55	12.87	40	70	54.99	10.29
Cl	mg/L	18.90	68.70	43.13	15.09	35.90	70.40	51.27	11.58
NO_3	mg/L	0.25	3	1.64	0.99	0.20	0.90	0.51	0.18
PO_4	mg/L	0.27	0.51	0.41	0.045	0.23	0.67	0.37	0.11
SO_4	mg/L	28	92.50	49.8	14.47	50.80	92.50	66.32	12.36
Al	mg/L	0.01	0.13	0.07	0.04	<0.001	0.35	0.07	0.08
Ba	mg/L	0.02	0.09	0.06	0.02	0.02	0.04	0.03	0.01
Cu	mg/L	0.009	0.04	0.013	0.001	0.012	0.07	0.035	0.013
Fe	mg/L	0.039	0.30	0.14	0.05	0.041	0.22	0.087	0.05
Mn	mg/L	<0.001	<0.001	<0.001	<0.001	<0.001	<0.001	<0.001	<0.001
Zn	mg/L	<0.001	<0.001	<0.001	<0.001	0.01	0.04	0.03	0.01

Table 3S Results of physiochemical and metals parameters of the water sample sites in Group B (SP8, SP10, SP12 and SP14) during summer and winter.

Parameters	Unit	Summer				Winter			
		Min	Max	Mean	SD	Min	Max	Mean	SD
pH	--	7.22	7.98	7.69	0.22	7.40	8.01	7.74	0.17
Total Alkalinity	mg/L	240	500	334.94	62.59	246	425	327.81	39.71
EC	µS/cm	1058	3098.	2007	691	2005	4480	3109	763
TDS	mg/L	635	1859	1204	415	1203	2688	1865	458
NH_3	mg/L	0.68	19	2.74	4.09	0.68	4	2.47	0.81
BOD	mg/L	3	20	13.94	4.84	2	19	14.19	4.70
COD	mg/L	12	54	29.50	12.43	16	40	30.72	7.33
Ca	mg/L	58	130.40	80.05	28.20	58	267.13	112.67	55.69
K	mg/L	10	62	20.81	12.85	7.05	30	13.53	6.05
Mg	mg/L	15	50.10	31.72	8.70	28.40	48.98	36.81	6.50
Na	mg/L	30.40	351	179.72	96.93	95	405	247.69	113.88
Cl	mg/L	94.80	360	182.20	100.47	94	593.10	252.04	143.69
NO_3	mg/L	0.11	8	4.43	3.13	0.11	9.10	4.68	3.61
PO_4	mg/L	0.48	1.5	0.9	0.05	0.21	2.35	0.68	0.61
SO_4	mg/L	92.50	520	230.09	152.51	100.5	648.5	297.22	161.86
Al	mg/L	0.03	1.51	0.91	0.26	0.03	0.20	0.12	0.05
Ba	mg/L	0.03	0.08	0.06	0.01	0.03	0.07	0.05	0.01
Cu	mg/L	<0.01	0.08	0.03	0.03	0.01	0.04	0.02	0.01
Fe	mg/L	0.06	0.30	0.14	0.08	0.06	0.42	0.22	0.13
Mn	mg/L	<0.01	<0.01	<0.01	<0.01	<0.01	<0.01	<0.01	<0.01
Zn	mg/L	<0.01	<0.01	<0.01	<0.01	0.01	0.04	0.03	0.01

Table 4S Results of physiochemical and metals parameters of the water sample sites in Group C (SP7, SP9, SP11, SP13 and SP15) during summer and winter

Parameters	Unit	Summer				Winter			
		Min	Max	Mean	SD	Min	Max	Mean	SD
pH	--	7.55	7.97	7.74	0.12	7.26	7.96	7.72	0.19
Total Alkalinity	mg/L	311	586	430.40	76.60	280.00	542	413.91	78.07
EC	μS/cm	1375	9833.33	4662.48	2705.75	2016.67	9800	3700.31	2072.63
TDS	mg/L	825	5900	2797.49	1623.45	1210	5880	2220.19	1243.58
NH_3	mg/L	1.40	7	3.71	1.55	13.90	31	17.71	4.43
BOD	mg/L	13	94	38.48	23.27	5	58	23.99	14.79
COD	mg/L	33	161	68.52	34.02	14.00	80	41.89	16.70
Ca	mg/L	70.18	186	122.82	26.40	85.33	277.88	171.24	63.46
K	mg/L	11	78	30.81	21.31	10	145	45.15	47.95
Mg	mg/L	20.10	366.4	110.43	127.81	19.10	345	72.65	85.35
Na	mg/L	148	1452	607.07	447.38	187	1400	452.42	327.43
Cl	mg/L	152	2280	641.02	657.76	268	2267	612.66	541.40
NO_3	mg/L	0.11	9.10	2.77	3.12	0.15	9.10	2.32	2.30
PO_4	mg/L	0.11	3.10	1.28	1.00	0.30	1.50	0.70	0.39
SO_4	mg/L	155	1246.74	605.58	372.44	234.50	1237	537.34	260.84
Al	mg/L	0.02	0.27	0.13	0.09	0.02	0.24	0.11	0.10
Ba	mg/L	0.04	0.08	0.06	0.01	0.03	0.08	0.05	0.01
Cu	mg/L	0.01	0.06	0.02	0.01	0.01	0.05	0.03	0.01
Fe	mg/L	0.03	0.98	0.23	0.23	0.03	0.27	0.13	0.08
Mn	mg/L	0.01	0.04	0.02	0.001	0.01	0.06	0.03	0.02
Zn	mg/L	0.01	0.011	0.01	0.001	0.01	0.02	0.01	0.001

Table 5S Fungal counts in different samples collected from canals and drains during summer and winter.

Samples group	Samples points	Summer (CFU/mL)			Winter (CFU/mL)		
		Min	Max	Average	Min	Max	Average
A	SP1	10	15	12±2	5	8	6±2
	SP2	24	38	34±6	10	12	11±1
	SP3	12	32	18±8	8	12	10±2
	SP4	8	33	27±11	3	18	12±8
	SP5	24	31	28±3	12	19	16±4
	SP6	13	76	28±27	6	30	15±13
C	SP7	41	48	44±3	22	28	25±4
B	SP8	63	73	63±10	30	34	31±2
C	SP9	43	55	48±5	21	28	24±4
B	SP10	49	56	53±4	27	31	28±2
C	SP11	22	68	51±17	11	29	22±9
B	SP12	9	65	46±22	6	18	13±6
C	SP13	52	65	57±6	31	33	32±1
B	SP14	20	31	24±5	9	22	17±7
C	SP15	50	71	61±8	32	35	33±2

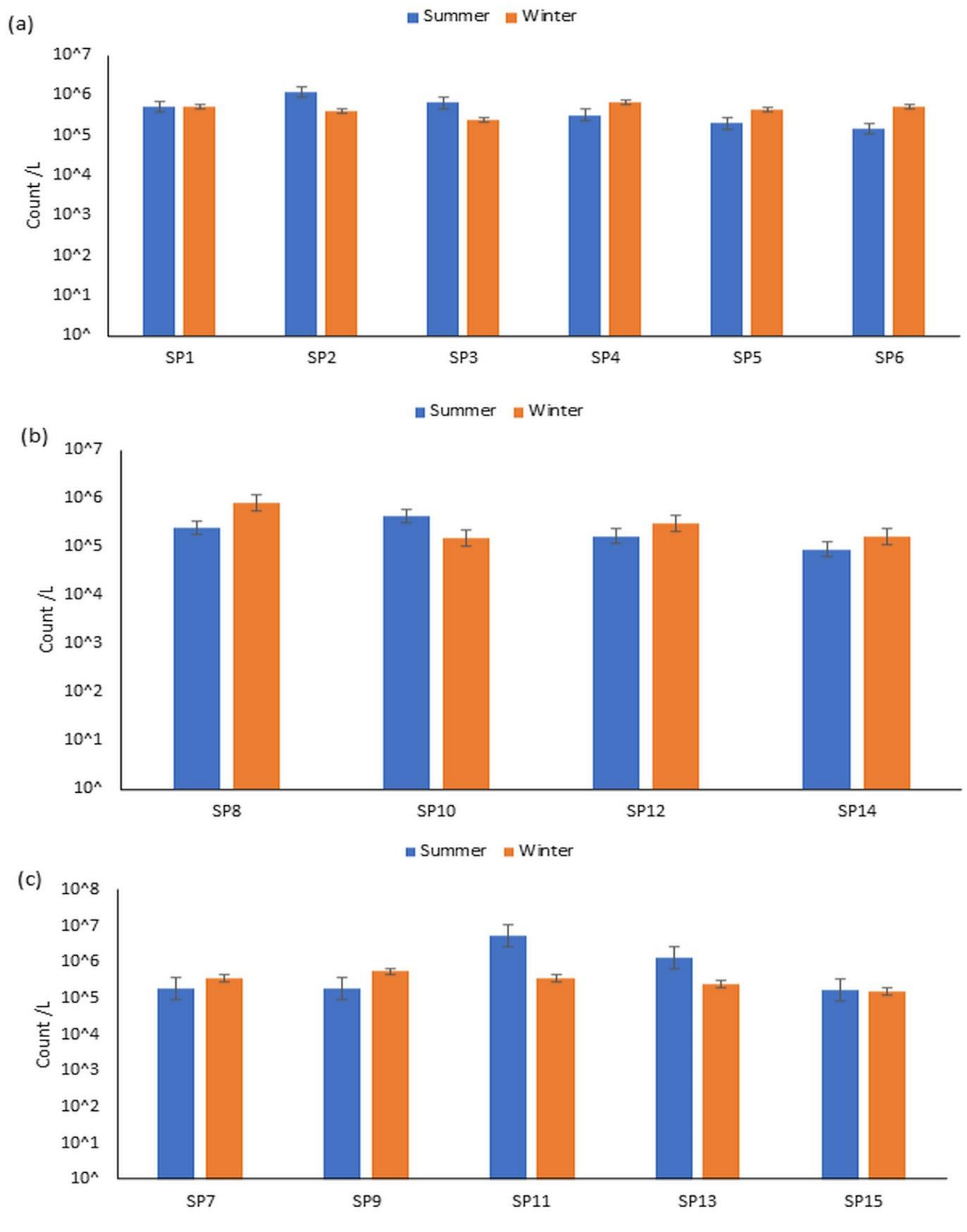

Fig. 6S Algae counts results for all collected samples during winter and summer.

References

1. Jin, R. *et al.* Magnetic core/shell Fe3O4/Au nanoparticles for studies of quinolones binding to protein by fluorescence spectroscopy. *Luminescence* **31**, 499–506 (2016).

2. Lesiak, B. *et al.* Surface Study of Fe3O4 Nanoparticles Functionalized With Biocompatible Adsorbed Molecules. *Front. Chem.* **7**, (2019).

3. Sangeetha, J., Thomas, S., Arutchelvi, J., Doble, M. & Philip, J. Functionalization of iron oxide nanoparticles with biosurfactants and biocompatibility studies. *J. Biomed. Nanotechnol.* **9**, 751–764 (2013).
